# Taxonomic and faunistic notes on the genus *Trichotichnus* from Korea (Coleoptera, Carabidae, Harpalinae, Harpalini)

**DOI:** 10.3897/BDJ.10.e83804

**Published:** 2022-06-06

**Authors:** Dooyoung Kim, Sang Jae Suh

**Affiliations:** 1 School of Applied Biosciences, Kyungpook National University, Daegu, Republic of Korea School of Applied Biosciences, Kyungpook National University Daegu Republic of Korea; 2 Institute of Plant Medicine, Kyungpook National University, Daegu, Republic of Korea Institute of Plant Medicine, Kyungpook National University Daegu Republic of Korea

**Keywords:** ground beetles, morphology, new record, taxonomy

## Abstract

**Background:**

*Trichotichnus* Morawitz, 1863 is a genus of the subtribe Harpalina, comprising more than 260 species worldwide. In Korea, nine species of the nominotypical subgenus are listed in the Korean catalogue (National Institute of Biological Resources 2019), while seven species of the nominotypical subgenus are listed to occur in Korea according to the Palaearctic catalogue (Kataev and Wrase 2017). Therefore, the checklist of the Korean species of the genus *Trichotichnus* needs to be revised.

**New information:**

In the course of taxonomic studies on *Trichotichnus* species from Korea, two unrecorded species are identified: Trichotichnus (Bottchrus) nanus and Trichotichnus (Trichotichnus) vespertinus. In addition, the distribution of a poorly known species, T. (T.) miser, is firstly recognized in the southern part of Korea. Along with the description of the newly-recorded species, a checklist of the Korean species of the genus *Trichotichnus* is revised. As for the checklist, the distribution of T. (Iridessus) lucidus and T. (T.) leptopus in Korea is re-discussed, and T. (T.) leptopus is excluded from the Korean fauna. Lastly, additional distribution records for the following species are provided: T. (I.) lucidus, T. (T.) longitarsis, T. (T.) nishioi, and T. (T.) noctuabundus.

## Introduction

*Trichotichnus* Morawitz, 1863 is a genus of the subtribe Harpalina, comprising more than 260 species worldwide (Kataev 2020). In Korea, the preliminary list of *Trichotichnus* was given by Kwon and Lee (1986), with a record of five species. Thereafter, this genus was partially studied by Paik (1995) and Paik (1997), and was later reviewed by Moon and Paik (2006c), along with their revisional study of Harpalini in Korea (Moon and Paik 2006a, Moon and Paik 2006b, Moon and Paik 2006c, Moon and Paik 2006d). In their study, nine species within the nominotypical subgenus were consequently recognized: Trichotichnus (Trichotichnus) congruus Motschulsky, 1866, T. (T.) coruscus (Tschitschérine, 1895), T. (T.) gerni Noonan, 1985, T. (T.) leptopus (Bates, 1883), T. (T.) longitarsis Morawitz, 1863, T. (T.) lucidus (Morawitz, 1863), T. (T.) nishioi Habu, 1961, T. (T.) noctuabundus Habu, 1954, and T. (T.) vicinus (Tschitschérine, 1897), and also a key was provided for the identification. Amongst the recorded species, the distribution of *T.lucidus* and *T.leptopus* in Korea was left to be unclear by the absence of the Korean specimen (Moon and Paik 2006c), but all the aforementioned species have been listed in the Korean catalogue until today (National Institute of Biological Resources 2019). In contrast, in the Palaearctic catalogue (Kataev and Wrase 2017), the Korean distribution record for *T.lucidus* and *T.leptopus* was excluded, based on their unclear distribution record, noted by Moon and Paik (2006c), and *T.miser* (Tschitschérine, 1897) was added, based on the specimen from North Korea. In case of *T.gerni*, it was synonymized with *Chydaeusandrewesiandrewesi* Schauberger, 1932 (Kataev and Schmidt 2006). As a result, the Palaearctic catalogue included the following seven *Trichotichnus* species to occur in the Korean fauna: T. (T.) congruus, T. (T.) coruscus, T. (T.) longitarsis, T. (T.) miser, T. (T.) nishioi, T. (T.) noctuabundus, and T. (T.) vicinus.

In this study, the distribution of *T.lucidus* and *T.leptopus* in Korea is re-discussed. First, the distribution of *T.lucidus* in Korea is confirmed. The specimen of *T.lucidus* was recently recorded by Kwon et al. (2018) from Jejudo Island, Korea and it was re-examined by the authors. Second, *T.leptopus* (Bates, 1883), which was first recorded in Korea by Yano (1941), is excluded from the Korean checklist, based on its distribution range. *T.leptopus* possesses a reduced hind wing and is known to be distributed in Kwantô District of Tochigi Prefecture, central Japan. Due to the reduced hind wing, *T.leptopus* and allied species (the so-called *leptopus*-group) have a localized distribution (Morita 1997). Moon and Paik (2006c) mentioned that the record of *T.leptopus* by Yano might be a misidentification of *T.coruscus*. Also, Dr. S. Morita mentioned that the record of *T.leptopus* in Korea is evidently a misidentification (personal communication). Considering that *T.leptopus* has a localized distribution, it might be reasonable to exclude this species from the Korean checklist.

Furthermore, the authors identified two species, which are new to the Korean fauna: Trichotichnus (Bottchrus) nanus Habu, 1954 and Trichotichnus (Trichotichnus) vespertinus Habu, 1954. In addition, Trichotichnus (Trichotichnus) miser (Tschitschérine, 1897), which is a poorly known species, was firstly recognized in the southern part of Korea. Consequently, a total of ten species are recognized to occur in Korea. Along with the description of the three species above, an updated checklist of Korean *Trichotichnus* species is provided.

## Materials and methods

The specimens were hand-collected at the collection sites and preserved in 95% ethanol or in dried condition. For the observation of the genital apparatus, 10% potassium hydroxide (KOH) solution was used for clearing. The genital apparatus was stored in a microtube with glycerine after observation. The examined specimens, except the specimen of T. (I.) lucidus, are deposited in the Systematic Entomology Laboratory, College of Agriculture and Life Sciences, Kyungpook National University (Daegu, Korea).

The habitus and male genital apparatus were observed and photographed under a stereoscopic microscope (Olympus SZX 16) and the female genital apparatus under a compound microscope (Olympus BX 50). The terminology used is based on Habu (1973) and Kataev and Schmidt (2017).

### Measurements

Body length measured from the anterior margin of the clypeus to the apex of elytra; head width (HW) measured including eye; pronotum width (PW) measured along the widest point; basal width of pronotum (PbW) measured the distance between the basal angles; pronotum length (PL) measured along the median line; elytra width (EW) measured along the widest point; elytra length (EL) measured from the basal border near scutellum to the apex of elytra; length of metepisternum (ML) measured along the outer margin; width of metepisternum (MW) measured along the anterior margin.

## Taxon treatments

### Trichotichnus

Morawitz, 1863

C31DF917-2F78-5875-B704-43F958639869

Trichotichnus Trichotichnus (Trichotichnus) longitarsis Morawitz, 1863Morawitz 1863: 63.

#### Diagnosis

The genus *Trichotichnus* can be distinguished from other genera by the following characteristics (Habu 1973, Kataev 2020): frontal impression extending towards margin of eye, mentum with developed tooth and with narrow epilobe, paraglossa glabrous; pronotum without posterior marginal seta; elytra smooth, often iridescent, interval 3 usually with one dorsal setigerous pore on stria 2 and without setigerous pore on intervals 5 and 7, elytral intervals without or with microsculpture consisting of transverse meshes; abdominal sternites mostly without extra setae; protibia with one to four (usually two or three) preapical spines on outer margin, apical spur slender, not dentate, metafemur with two (rarely three) setae on hind margin, male pro- and mesotarsus with ventral biseriate vestiture; median lobe of aedeagus with apical orifice in dorsal position or shifted to left, with or without apical capitulum; hemisternite with one to five setae or small spines apically.

### Trichotichnus (Bottchrus) nanus

Habu, 1954

A85123F9-44B9-59F8-9244-A7DB7168A23D

Trichotichnus nanus Habu, 1954 - Habu 1954c: 255.
Trichotichnus (Trichotichnus) nanus : Habu, 1961 - Habu 1961: 144.
Trichotichnus (Pseudotrichotichnus) nanus : Habu, 1973 - Habu 1973: 226.
Trichotichnus (Bottchrus) nanus : Kataev and Wrase, 2017 - Kataev and Wrase 2017: 558.

#### Materials

- **Type status:** Other material. **Occurrence:** sex: 5 males, 4 females; lifeStage: adult; **Taxon:** scientificName: Trichotichnus (Bottchrus) nanus; order: Coleoptera; family: Carabidae; genus: Trichotichnus; subgenus: Bottchrus; specificEpithet: *nanus*; vernacularName: 꼬마윤머리먼지벌레; **Location:** country: Korea; stateProvince: Daegu-si; locality: Suseong-gu, Beommul-dong, Jinbatgol, Coll. Dooyoung Kim; verbatimLatitude: 35°48'36.1"N; verbatimLongitude: 128°39'44.5"E; **Event:** samplingProtocol: under the leaf litter; eventDate: 1.VII.2021; **Record Level:** basisOfRecord: PreservedSpecimen
- **Type status:** Other material. **Occurrence:** sex: 3 males; lifeStage: adult; **Taxon:** scientificName: Trichotichnus (Bottchrus) nanus; order: Coleoptera; family: Carabidae; genus: Trichotichnus; subgenus: Bottchrus; specificEpithet: *nanus*; vernacularName: 꼬마윤머리먼지벌레; **Location:** country: Korea; stateProvince: Daegu-si; locality: Suseong-gu, Beommul-dong, Jinbatgol, Coll. Dooyoung Kim; verbatimLatitude: 35°48'36.1"N; verbatimLongitude: 128°39'44.5"E; **Event:** samplingProtocol: under the leaf litter; eventDate: 10.VII.2021; **Record Level:** basisOfRecord: PreservedSpecimen
- **Type status:** Other material. **Occurrence:** sex: 1 male; lifeStage: adult; **Taxon:** scientificName: Trichotichnus (Bottchrus) nanus; order: Coleoptera; family: Carabidae; genus: Trichotichnus; subgenus: Bottchrus; specificEpithet: *nanus*; vernacularName: 꼬마윤머리먼지벌레; **Location:** country: Korea; stateProvince: Daegu-si; locality: Suseong-gu, Hwanggeum-dong, Duribong, Coll. Dooyoung Kim; verbatimLatitude: 35°50'17.3"N; verbatimLongitude: 128°38'31.2"E; **Event:** samplingProtocol: under the leaf litter; eventDate: 26.V.2018; **Record Level:** basisOfRecord: PreservedSpecimen
- **Type status:** Other material. **Occurrence:** sex: 1 male, 1 female; lifeStage: adult; **Taxon:** scientificName: Trichotichnus (Bottchrus) nanus; order: Coleoptera; family: Carabidae; genus: Trichotichnus; subgenus: Bottchrus; specificEpithet: *nanus*; vernacularName: 꼬마윤머리먼지벌레; **Location:** country: Korea; stateProvince: Daegu-si; locality: Suseong-gu, Hwanggeum-dong, Duribong, Coll. Dooyoung Kim; verbatimLatitude: 35°50'17.3"N; verbatimLongitude: 128°38'31.2"E; **Event:** samplingProtocol: under the leaf litter; eventDate: 31.V.2018; **Record Level:** basisOfRecord: PreservedSpecimen
- **Type status:** Other material. **Occurrence:** sex: 1 female; lifeStage: adult; **Taxon:** scientificName: Trichotichnus (Bottchrus) nanus; order: Coleoptera; family: Carabidae; genus: Trichotichnus; subgenus: Bottchrus; specificEpithet: *nanus*; vernacularName: 꼬마윤머리먼지벌레; **Location:** country: Korea; stateProvince: Daegu-si; locality: Suseong-gu, Hwanggeum-dong, Duribong, Coll. Dooyoung Kim; verbatimLatitude: 35°50'17.3"N; verbatimLongitude: 128°38'31.2"E; **Event:** samplingProtocol: under the leaf litter; eventDate: 6.VI.2018; **Record Level:** basisOfRecord: PreservedSpecimen
- **Type status:** Other material. **Occurrence:** sex: 3 females; lifeStage: adult; **Taxon:** scientificName: Trichotichnus (Bottchrus) nanus; order: Coleoptera; family: Carabidae; genus: Trichotichnus; subgenus: Bottchrus; specificEpithet: *nanus*; vernacularName: 꼬마윤머리먼지벌레; **Location:** country: Korea; stateProvince: Daegu-si; locality: Suseong-gu, Hwanggeum-dong, Duribong, Coll. Dooyoung Kim; verbatimLatitude: 35°50'17.3"N; verbatimLongitude: 128°38'31.2"E; **Event:** samplingProtocol: under the leaf litter; eventDate: 15.V.2019; **Record Level:** basisOfRecord: PreservedSpecimen
- **Type status:** Other material. **Occurrence:** sex: 1 male; lifeStage: adult; **Taxon:** scientificName: Trichotichnus (Bottchrus) nanus; order: Coleoptera; family: Carabidae; genus: Trichotichnus; subgenus: Bottchrus; specificEpithet: *nanus*; vernacularName: 꼬마윤머리먼지벌레; **Location:** country: Korea; stateProvince: Daegu-si; locality: Suseong-gu, Hwanggeum-dong, Duribong, Coll. Dooyoung Kim; verbatimLatitude: 35°50'17.3"N; verbatimLongitude: 128°38'31.2"E; **Event:** samplingProtocol: under the leaf litter; eventDate: 3.V.2021; **Record Level:** basisOfRecord: PreservedSpecimen
- **Type status:** Other material. **Occurrence:** sex: 1 male; lifeStage: adult; **Taxon:** scientificName: Trichotichnus (Bottchrus) nanus; order: Coleoptera; family: Carabidae; genus: Trichotichnus; subgenus: Bottchrus; specificEpithet: *nanus*; vernacularName: 꼬마윤머리먼지벌레; **Location:** country: Korea; stateProvince: Daegu-si; locality: Suseong-gu, Hwanggeum-dong, Duribong, Coll. Dooyoung Kim; verbatimLatitude: 35°50'17.3"N; verbatimLongitude: 128°38'31.2"E; **Event:** samplingProtocol: under the leaf litter; eventDate: 22.VI.2021; **Record Level:** basisOfRecord: PreservedSpecimen
- **Type status:** Other material. **Occurrence:** sex: 1 male; lifeStage: adult; **Taxon:** scientificName: Trichotichnus (Bottchrus) nanus; order: Coleoptera; family: Carabidae; genus: Trichotichnus; subgenus: Bottchrus; specificEpithet: *nanus*; vernacularName: 꼬마윤머리먼지벌레; **Location:** country: Korea; stateProvince: Daegu-si; locality: Suseong-gu, Hwanggeum-dong, Duribong, Coll. Dooyoung Kim; verbatimLatitude: 35°50'17.3"N; verbatimLongitude: 128°38'31.2"E; **Event:** samplingProtocol: under the leaf litter; eventDate: 30.VI.2021; **Record Level:** basisOfRecord: PreservedSpecimen

#### Description

Body length: 6.0–7.1 mm, width: 2.6–2.8 mm.

**Figure 1.**
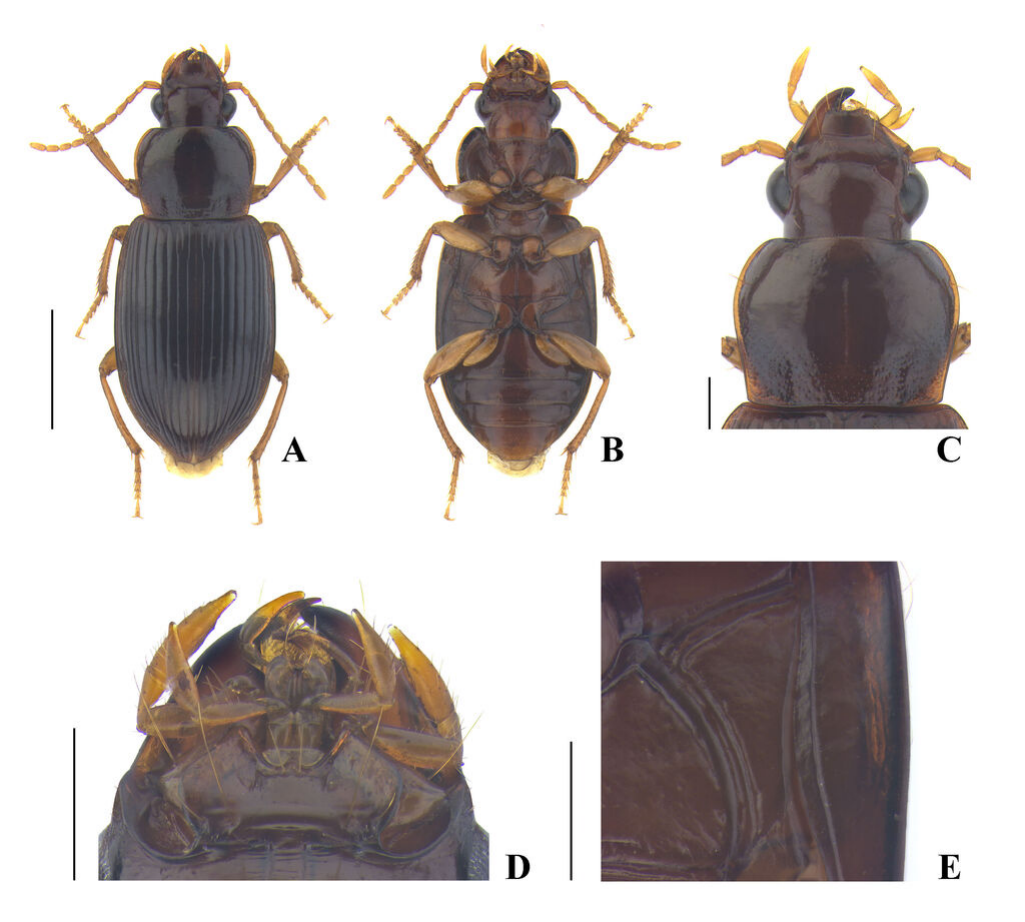
Trichotichnus (Bottchrus) nanus Habu, 1954, male: **A** habitus, dorsal view; **B** ditto, ventral view; **C** head and pronotum, dorsal view; **D** head, ventral view; **E** metepisternum. Scale bars: 2 mm (**A, B**); 0.5 mm (**C**–**E**).

**Coloration** (Fig. 1A, B) shiny, reddish black or brownish black, elytra slightly iridescent; antennae, maxillary palpi, labial palpi, lateral margin of pronotum, and legs yellowish brown; labrum and mandibles brownish black or reddish black; apex of each mandible black; ventral side overall reddish brown.

**Head** (Fig. 1C, D) convex and glabrous; eye prominent, distinctly convex; tempora flat, about one-third as long as eye; mandible narrowly rounded at apex, not truncate; anterior margin of labrum distinctly, sometimes slightly concave; anterior margin of clypeus slightly emarginate; frontal impression deep throughout, clearly reaching margin of eye and further extending posteriorly to the level of hind margin of eye; supraorbital seta located slightly behind the level of hind margin of eye; microsculpture invisible on disc, consisting of faint isodiametric meshes between supraorbital seta and hind margin of eye; postgena finely ciliate; mentum and submentum separated by transverse suture medially and fused laterally; mentum with distinct tooth, which is narrowly rounded at apex, epilobe gently widened anteriorly, slightly or sometimes distinctly projected beyond lateral lobe; submentum with a long seta and a short seta on each side; ligula narrow, apex truncate, with two apical setae; paraglossa moderately wide, rounded at apex, separated from ligula by somewhat narrow notch; penultimate maxillary palpomere about half as long as apical palpomere; penultimate labial palpomere slightly shorter than apical palpomere; antenna short, extending slightly behind basal margin of pronotum.

**Pronotum** (Fig. 1C) convex anteriorly, somewhat flat posteriorly, wider than long, widest near 2/5 from apex, PbW longer than PL; apical margin slightly emarginate, completely bordered; apical angle rounded, very slightly protruding anteriorly; lateral margin with very weak sinuation or without sinuation before basal angle; lateral seta located before widest point, about 1/4 from apex; basal margin almost straight, faintly bisinuate, distinctly longer than apical margin, completely bordered; basal angle obtuse, almost angulate, rounded at tip, not protruding laterally; disc glabrous; median line shallow or deep, interrupted near base, not reaching apical and basal border; anterior impression faint; basal fovea shallow to moderately deep, straight or weakly curved, slightly tilted outwards; basal area punctate through lateral to median area, usually densely punctate in basal fovea and sparsely punctate in lateral area; lateral area sparsely punctate from base to mid-point; apical area and disc almost smooth; microsculpture invisible on disc. PW/HW = 1.37–1.44, mean 1.40; PW/PL = 1.28–1.38, mean 1.33; PW/PbW = 1.15–1.25, mean 1.19, in ten males and ten females.

**Elytra** convex, widest before middle; basal edge evenly curved, forming an obtuse angle with lateral margin; humerus with indistinct blunted tooth visible from behind; apical sinuation very weak; intervals somewhat flat, slightly convex near lateral area and apex, without punctation; setigerous pore of interval 3 located between 1/2 to 3/5 from base; striae moderately deep; parascutellar striole not long, sometimes quite short; marginal umbilicate series with 7–10 pores (usually 8 pores) in anterior group and 9–12 pores (usually 10 pores) in posterior group; microsculpture invisible on disc. Hind wing fully developed. EW/PW = 1.27–1.35, mean 1.31; EL/EW = 1.48–1.55, mean 1.52, in 10 males and 10 females.

**Ventral side** (Fig. 1B, E) convex; prosternum covered with very short and fine pubescence; median area of metasternum and median area of abdominal sternites III–VII more sparsely pubescence than prosternum; pro-, meso-, metepisternum, and metasternum smooth, without punctation, sometimes faintly rugose on metepisternum and lateral area of metasterum; metepisternum (Fig. 1E) distinctly longer than wide (ML/MW = 1.37–1.53, mean 1.47, in seven males and six females); anterior and inner margins bordered; apical margin of abdominal sternite VII slightly emarginate and with two pairs of setae in both sexes.

**Legs**. Metacoxa with two setae; metafemur with two setae in hind margin; metatarsomere 1 slightly shorter than metatarsomere 2 and 3 combined, about 1.6 times as long as metatarsomere 2; metatarsomere 5 about 1.1 times as long as metatarsomere 1; tarsomere 5 with two setae in each ventrolateral margin.

**Figure 2.**
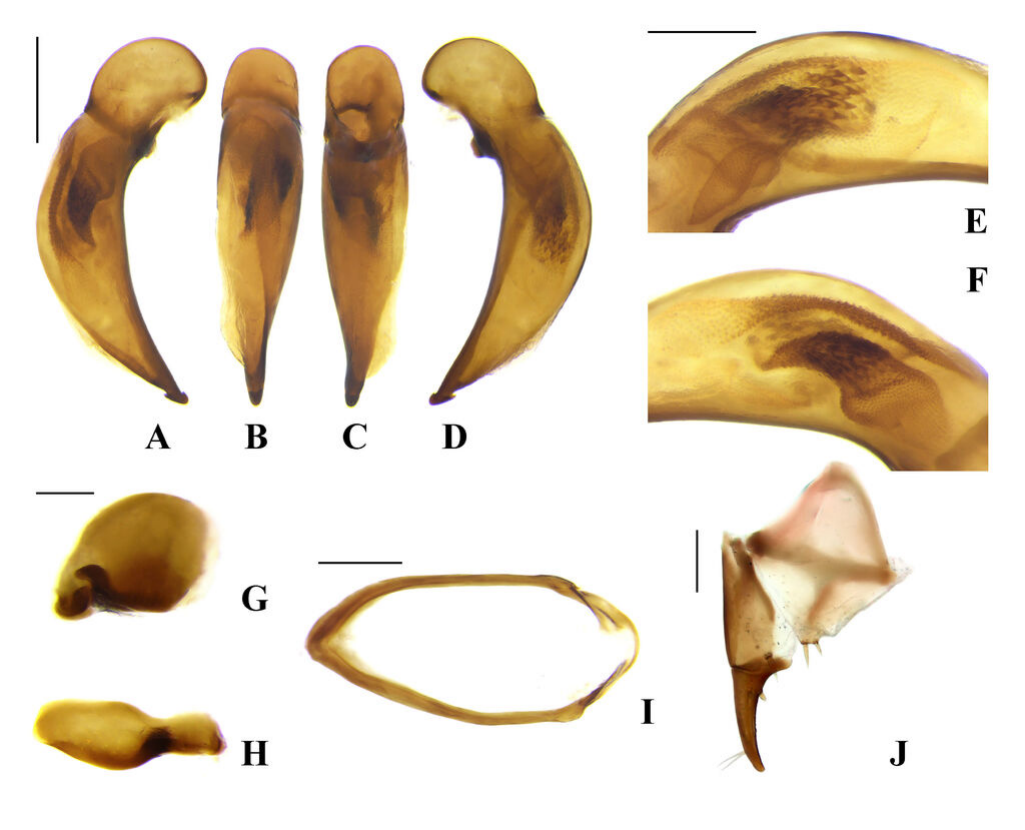
Male and female genitalia of Trichotichnus (Bottchrus) nanus Habu, 1954: **A** median lobe of aedeagus, right lateral view; **B** ditto, dorsal view; **C** ditto, ventral view; **D** ditto, left lateral view; **E** sclerotic armature of internal sac of aedeagus, left lateral view; **F** ditto, right lateral view; **G** left paramere; **H** right paramere; **I** male sternite Ⅸ; **J** female genitalia. Scale bars: 0.5 mm (**A**–**D, I**); 0.3 mm (**E, F, J**); 0.2 mm (**G, H**).

**Male genitalia** (Fig. 2A–I). Median lobe of aedeagus (Fig. 2A–F) gently curved in lateral view, almost straight in dorsal view; terminal lamella narrow, distinctly longer than wide; apical capitulum present, distinctly protruding ventrally and dorsally, posterior margin of apical capitulum in dorsal view somewhat distinctly emarginate; median part of internal sac in left lateral view (Fig. 2E) consisting of numerous small spines; internal sac in right lateral view (Fig. 2F) large hook-shaped, consisting of numerous, dense small spines; ventral side of median lobe not bordered; parameres as in Fig. 2G, H; sternite IX (Fig. 2I) not explanate basally.

**Female genitalia** (Fig. 2J). Hemisternite with two spines apically; basal stylomere with one preapical spine on external margin; apical stylomere slender with two setae subapically, inner margin almost straight from base to point near subapical setae and slightly curved apically, outer margin gently curved from base to apex, dorsal and ventral outer margins with one small distinct spine, respectively.

#### Diagnosis

This species can be distinguished from others by the following characters: overall reddish black or brownish black coloration; postgena finely ciliate, penultimate maxillary palpomere distinctly short, about half as long as apical palpomere; pronotum with narrowly rounded basal angle, apical margin completely bordered, lateral margin with faint or without sinuation before basal angle; hind wing fully developed; internal sac of aedeagus in right lateral view (Fig. 2F) large hook-shaped, posterior margin of apical capitulum in dorsal view somewhat distinctly emarginate.

#### Distribution

Korea: South (new record); Japan: Kyushu (Habu 1973), Honshu (Mori 2016).

#### Ecology

**Figure 3.**
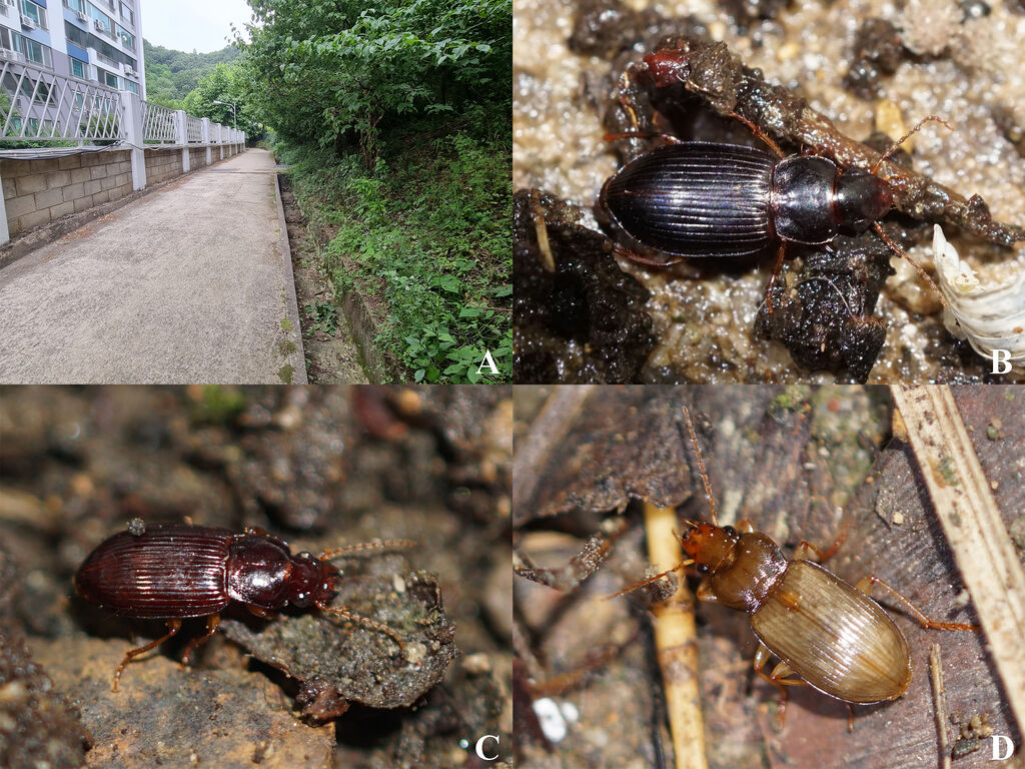
Observation environment and living examples: **A** observation environment of T. (B.) nanus Habu, T. (T.) miser (Tschitschérine), and T. (T.) vespertinus Habu; **B** adult of T. (T.) miser (Tschitschérine); **C** adult of T. (B.) nanus Habu; **D** immature adult of T. (T.) vespertinus Habu.

All the individuals were observed from May to early July and mating behavior was observed in late June. None of the immature adults was observed during the study. Most individuals were observed under the leaf litter in the deciduous forest or at the bottom of the drainage channel, located in the surroundings of the deciduous forest (Fig. 3A, C).

#### Notes

Even though the description of T. (B.) nanus by Habu (1961) and Habu (1973) indicates that there are 'no teeth (spines)' in the internal sac, all of the examined Korean male specimens had spines in the internal sac of the aedeagus. Dr. S. Morita kindly sent photography of the aedeagus of the specimen from Osaka (Japan) to the authors for comparison, and it also clearly possessed the spines in the internal sac. The authors conclude that there might be a variation in the presence of the spine in the internal sac of the aedeagus. This species was previously only known to occur in Japan, but a new distribution record is added to Korea.

### Trichotichnus (Trichotichnus) miser

(Tschitschérine, 1897)

5E58735F-CB90-5624-A0CD-4E4281F73595

Harpalus miser Tschitschérine, 1897 - Tschitschérine 1897: 57.
Asmerinx misera : Tschitschérine, 1898 - Tschitschérine 1898: 184.
Trichotichnus (Trichotichnus) miser : Noonan, 1985 - Noonan 1985: 68.

#### Materials

- **Type status:** Other material. **Occurrence:** sex: 1 female; lifeStage: adult; **Taxon:** scientificName: Trichotichnus (Trichotichnus) miser; order: Coleoptera; family: Carabidae; genus: Trichotichnus; subgenus: Trichotichnus; specificEpithet: miser; scientificNameAuthorship: (Tschitschérine, 1897); vernacularName: 둥근가슴윤머리먼지벌레; **Location:** country: Korea; stateProvince: Daegu-si; locality: Dalseong-gun, Gachang-myeon, Ju-ri, Mt. Choejeongsan, Coll. Dooyoung Kim; verbatimLatitude: 35°44'34.9"N; verbatimLongitude: 128°36'51.0"E; **Event:** samplingProtocol: under the leaf litter; eventDate: 31.VII.2021; **Record Level:** basisOfRecord: PreservedSpecimen
- **Type status:** Other material. **Occurrence:** sex: 1 male, 1 female; lifeStage: adult; **Taxon:** scientificName: Trichotichnus (Trichotichnus) miser; order: Coleoptera; family: Carabidae; genus: Trichotichnus; subgenus: Trichotichnus; specificEpithet: *miser*; scientificNameAuthorship: (Tschitschérine, 1897); vernacularName: 둥근가슴윤머리먼지벌레; **Location:** country: Korea; stateProvince: Daegu-si; locality: Dalseong-gun, Gachang-myeon, Ju-ri, Mt. Choejeongsan, Coll. Dooyoung Kim; verbatimLatitude: 35°44'34.9"N; verbatimLongitude: 128°36'51.0"E; **Event:** samplingProtocol: under the leaf litter; eventDate: 30.Ⅹ.2021; **Record Level:** basisOfRecord: PreservedSpecimen
- **Type status:** Other material. **Occurrence:** sex: 1 female; lifeStage: adult; **Taxon:** scientificName: Trichotichnus (Trichotichnus) miser; order: Coleoptera; family: Carabidae; genus: Trichotichnus; subgenus: Trichotichnus; specificEpithet: miser; scientificNameAuthorship: (Tschitschérine, 1897); vernacularName: 둥근가슴윤머리먼지벌레; **Location:** country: Korea; stateProvince: Daegu-si; locality: Suseong-gu, Beommul-dong, Jinbatgol, Coll. Dooyoung Kim; verbatimLatitude: 35°47'56.6"N; verbatimLongitude: 128°39'58.6"E; **Event:** samplingProtocol: under the leaf litter; eventDate: 4.Ⅴ.2018; **Record Level:** basisOfRecord: PreservedSpecimen
- **Type status:** Other material. **Occurrence:** sex: 1 male; lifeStage: adult; **Taxon:** scientificName: Trichotichnus (Trichotichnus) miser; order: Coleoptera; family: Carabidae; genus: Trichotichnus; subgenus: Trichotichnus; specificEpithet: miser; scientificNameAuthorship: (Tschitschérine, 1897); vernacularName: 둥근가슴윤머리먼지벌레; **Location:** country: Korea; stateProvince: Daegu-si; locality: Suseong-gu, Beommul-dong, Jinbatgol, Coll. Dooyoung Kim; verbatimLatitude: 35°48'03.9"N; verbatimLongitude: 128°40'02.2"E; **Event:** samplingProtocol: under the leaf litter; eventDate: 8.IX.2018; **Record Level:** basisOfRecord: PreservedSpecimen
- **Type status:** Other material. **Occurrence:** sex: 1 male, 2 females; lifeStage: adult; **Taxon:** scientificName: Trichotichnus (Trichotichnus) miser; order: Coleoptera; family: Carabidae; genus: Trichotichnus; subgenus: Trichotichnus; specificEpithet: miser; scientificNameAuthorship: (Tschitschérine, 1897); vernacularName: 둥근가슴윤머리먼지벌레; **Location:** country: Korea; stateProvince: Daegu-si; locality: Suseong-gu, Beommul-dong, Jinbatgol, Coll. Dooyoung Kim; verbatimLatitude: 35°47'56.6"N; verbatimLongitude: 128°39'58.6"E; **Event:** samplingProtocol: under the leaf litter; eventDate: 25.III.2021; **Record Level:** basisOfRecord: PreservedSpecimen
- **Type status:** Other material. **Occurrence:** sex: 1 male; lifeStage: adult; **Taxon:** scientificName: Trichotichnus (Trichotichnus) miser; order: Coleoptera; family: Carabidae; genus: Trichotichnus; subgenus: Trichotichnus; specificEpithet: miser; scientificNameAuthorship: (Tschitschérine, 1897); vernacularName: 둥근가슴윤머리먼지벌레; **Location:** country: Korea; stateProvince: Daegu-si; locality: Suseong-gu, Beommul-dong, Jinbatgol, Coll. Dooyoung Kim; verbatimLatitude: 35°47'56.6"N; verbatimLongitude: 128°39'58.6"E; **Event:** samplingProtocol: under the leaf litter; eventDate: 16.IV.2021; **Record Level:** basisOfRecord: PreservedSpecimen
- **Type status:** Other material. **Occurrence:** sex: 1 male; lifeStage: adult; **Taxon:** scientificName: Trichotichnus (Trichotichnus) miser; order: Coleoptera; family: Carabidae; genus: Trichotichnus; subgenus: Trichotichnus; specificEpithet: miser; scientificNameAuthorship: (Tschitschérine, 1897); vernacularName: 둥근가슴윤머리먼지벌레; **Location:** country: Korea; stateProvince: Daegu-si; locality: Suseong-gu, Jisan-dong, Joilgol, Coll. Dooyoung Kim; verbatimLatitude: 35°49'47.0"N; verbatimLongitude: 128°38'47.5"E; **Event:** samplingProtocol: under the leaf litter; eventDate: 1.VII.2021; **Record Level:** basisOfRecord: PreservedSpecimen
- **Type status:** Other material. **Occurrence:** sex: 2 males, 3 females; lifeStage: adult; **Taxon:** scientificName: Trichotichnus (Trichotichnus) miser; order: Coleoptera; family: Carabidae; genus: Trichotichnus; subgenus: Trichotichnus; specificEpithet: miser; scientificNameAuthorship: (Tschitschérine, 1897); vernacularName: 둥근가슴윤머리먼지벌레; **Location:** country: Korea; stateProvince: Daegu-si; locality: Suseong-gu, Hwanggeum-dong, Duribong, Coll. Dooyoung Kim; verbatimLatitude: 35°50'17.3"N; verbatimLongitude: 128°38'31.2"E; **Event:** samplingProtocol: under the leaf litter; eventDate: 28.III.2018; **Record Level:** basisOfRecord: PreservedSpecimen
- **Type status:** Other material. **Occurrence:** sex: 6 males, 2 females; lifeStage: adult; **Taxon:** scientificName: Trichotichnus (Trichotichnus) miser; order: Coleoptera; family: Carabidae; genus: Trichotichnus; subgenus: Trichotichnus; specificEpithet: miser; scientificNameAuthorship: (Tschitschérine, 1897); vernacularName: 둥근가슴윤머리먼지벌레; **Location:** country: Korea; stateProvince: Daegu-si; locality: Suseong-gu, Hwanggeum-dong, Duribong, Coll. Dooyoung Kim; verbatimLatitude: 35°50'17.3"N; verbatimLongitude: 128°38'31.2"E; **Event:** samplingProtocol: under the leaf litter; eventDate: 31.III.2018; **Record Level:** basisOfRecord: PreservedSpecimen
- **Type status:** Other material. **Occurrence:** sex: 1 male, 2 females; lifeStage: adult; **Taxon:** scientificName: Trichotichnus (Trichotichnus) miser; order: Coleoptera; family: Carabidae; genus: Trichotichnus; subgenus: Trichotichnus; specificEpithet: miser; scientificNameAuthorship: (Tschitschérine, 1897); vernacularName: 둥근가슴윤머리먼지벌레; **Location:** country: Korea; stateProvince: Daegu-si; locality: Suseong-gu, Hwanggeum-dong, Duribong, Coll. Dooyoung Kim; verbatimLatitude: 35°50'17.3"N; verbatimLongitude: 128°38'31.2"E; **Event:** samplingProtocol: under the leaf litter; eventDate: 12.IV.2018; **Record Level:** basisOfRecord: PreservedSpecimen
- **Type status:** Other material. **Occurrence:** sex: 1 male; lifeStage: adult; **Taxon:** scientificName: Trichotichnus (Trichotichnus) miser; order: Coleoptera; family: Carabidae; genus: Trichotichnus; subgenus: Trichotichnus; specificEpithet: miser; scientificNameAuthorship: (Tschitschérine, 1897); vernacularName: 둥근가슴윤머리먼지벌레; **Location:** country: Korea; stateProvince: Daegu-si; locality: Suseong-gu, Hwanggeum-dong, Duribong, Coll. Dooyoung Kim; verbatimLatitude: 35°50'17.3"N; verbatimLongitude: 128°38'31.2"E; **Event:** samplingProtocol: under the leaf litter; eventDate: 26.IV.2018; **Record Level:** basisOfRecord: PreservedSpecimen
- **Type status:** Other material. **Occurrence:** sex: 1 female; lifeStage: adult; **Taxon:** scientificName: Trichotichnus (Trichotichnus) miser; order: Coleoptera; family: Carabidae; genus: Trichotichnus; subgenus: Trichotichnus; specificEpithet: miser; scientificNameAuthorship: (Tschitschérine, 1897); vernacularName: 둥근가슴윤머리먼지벌레; **Location:** country: Korea; stateProvince: Daegu-si; locality: Suseong-gu, Hwanggeum-dong, Duribong, Coll. Dooyoung Kim; verbatimLatitude: 35°50'17.3"N; verbatimLongitude: 128°38'31.2"E; **Event:** samplingProtocol: under the leaf litter; eventDate: 10.Ⅴ.2018; **Record Level:** basisOfRecord: PreservedSpecimen
- **Type status:** Other material. **Occurrence:** sex: 1 male; lifeStage: adult; **Taxon:** scientificName: Trichotichnus (Trichotichnus) miser; order: Coleoptera; family: Carabidae; genus: Trichotichnus; subgenus: Trichotichnus; specificEpithet: miser; scientificNameAuthorship: (Tschitschérine, 1897); vernacularName: 둥근가슴윤머리먼지벌레; **Location:** country: Korea; stateProvince: Daegu-si; locality: Suseong-gu, Hwanggeum-dong, Duribong, Coll. Dooyoung Kim; verbatimLatitude: 35°50'17.3"N; verbatimLongitude: 128°38'31.2"E; **Event:** samplingProtocol: under the leaf litter; eventDate: 31.Ⅴ.2018; **Record Level:** basisOfRecord: PreservedSpecimen
- **Type status:** Other material. **Occurrence:** sex: 1 female; lifeStage: adult; **Taxon:** scientificName: Trichotichnus (Trichotichnus) miser; order: Coleoptera; family: Carabidae; genus: Trichotichnus; subgenus: Trichotichnus; specificEpithet: miser; scientificNameAuthorship: (Tschitschérine, 1897); vernacularName: 둥근가슴윤머리먼지벌레; **Location:** country: Korea; stateProvince: Daegu-si; locality: Suseong-gu, Hwanggeum-dong, Duribong, Coll. Dooyoung Kim; verbatimLatitude: 35°50'17.3"N; verbatimLongitude: 128°38'31.2"E; **Event:** samplingProtocol: under the leaf litter; eventDate: 23.VI.2019; **Record Level:** basisOfRecord: PreservedSpecimen
- **Type status:** Other material. **Occurrence:** sex: 1 male, 5 females; lifeStage: adult; **Taxon:** scientificName: Trichotichnus (Trichotichnus) miser; order: Coleoptera; family: Carabidae; genus: Trichotichnus; subgenus: Trichotichnus; specificEpithet: miser; scientificNameAuthorship: (Tschitschérine, 1897); vernacularName: 둥근가슴윤머리먼지벌레; **Location:** country: Korea; stateProvince: Daegu-si; locality: Suseong-gu, Hwanggeum-dong, Duribong, Coll. Dooyoung Kim; verbatimLatitude: 35°50'17.3"N; verbatimLongitude: 128°38'31.2"E; **Event:** samplingProtocol: under the leaf litter; eventDate: 10.III.2021; **Record Level:** basisOfRecord: PreservedSpecimen
- **Type status:** Other material. **Occurrence:** sex: 1 male, 1 female; lifeStage: adult; **Taxon:** scientificName: Trichotichnus (Trichotichnus) miser; order: Coleoptera; family: Carabidae; genus: Trichotichnus; subgenus: Trichotichnus; specificEpithet: miser; scientificNameAuthorship: (Tschitschérine, 1897); vernacularName: 둥근가슴윤머리먼지벌레; **Location:** country: Korea; stateProvince: Daegu-si; locality: Suseong-gu, Hwanggeum-dong, Duribong, Coll. Dooyoung Kim; verbatimLatitude: 35°50'17.3"N; verbatimLongitude: 128°38'31.2"E; **Event:** samplingProtocol: under the leaf litter; eventDate: 16.III.2021; **Record Level:** basisOfRecord: PreservedSpecimen
- **Type status:** Other material. **Occurrence:** sex: 1 male; lifeStage: adult; **Taxon:** scientificName: Trichotichnus (Trichotichnus) miser; order: Coleoptera; family: Carabidae; genus: Trichotichnus; subgenus: Trichotichnus; specificEpithet: miser; scientificNameAuthorship: (Tschitschérine, 1897); vernacularName: 둥근가슴윤머리먼지벌레; **Location:** country: Korea; stateProvince: Daegu-si; locality: Suseong-gu, Hwanggeum-dong, Duribong, Coll. Dooyoung Kim; verbatimLatitude: 35°50'17.3"N; verbatimLongitude: 128°38'31.2"E; **Event:** samplingProtocol: under the leaf litter; eventDate: 19.III.2021; **Record Level:** basisOfRecord: PreservedSpecimen
- **Type status:** Other material. **Occurrence:** sex: 2 females; lifeStage: adult; **Taxon:** scientificName: Trichotichnus (Trichotichnus) miser; order: Coleoptera; family: Carabidae; genus: Trichotichnus; subgenus: Trichotichnus; specificEpithet: miser; scientificNameAuthorship: (Tschitschérine, 1897); vernacularName: 둥근가슴윤머리먼지벌레; **Location:** country: Korea; stateProvince: Daegu-si; locality: Suseong-gu, Hwanggeum-dong, Duribong, Coll. Dooyoung Kim; verbatimLatitude: 35°50'17.3"N; verbatimLongitude: 128°38'31.2"E; **Event:** samplingProtocol: under the leaf litter; eventDate: 1.IV.2021; **Record Level:** basisOfRecord: PreservedSpecimen
- **Type status:** Other material. **Occurrence:** sex: 2 males; lifeStage: adult; **Taxon:** scientificName: Trichotichnus (Trichotichnus) miser; order: Coleoptera; family: Carabidae; genus: Trichotichnus; subgenus: Trichotichnus; specificEpithet: miser; scientificNameAuthorship: (Tschitschérine, 1897); vernacularName: 둥근가슴윤머리먼지벌레; **Location:** country: Korea; stateProvince: Daegu-si; locality: Suseong-gu, Hwanggeum-dong, Duribong, Coll. Dooyoung Kim; verbatimLatitude: 35°50'17.3"N; verbatimLongitude: 128°38'31.2"E; **Event:** samplingProtocol: under the leaf litter; eventDate: 17.VI.2021; **Record Level:** basisOfRecord: PreservedSpecimen
- **Type status:** Other material. **Occurrence:** sex: 2 females; lifeStage: adult; **Taxon:** scientificName: Trichotichnus (Trichotichnus) miser; order: Coleoptera; family: Carabidae; genus: Trichotichnus; subgenus: Trichotichnus; specificEpithet: miser; scientificNameAuthorship: (Tschitschérine, 1897); vernacularName: 둥근가슴윤머리먼지벌레; **Location:** country: Korea; stateProvince: Daegu-si; locality: Suseong-gu, Hwanggeum-dong, Duribong, Coll. Dooyoung Kim; verbatimLatitude: 35°50'17.3"N; verbatimLongitude: 128°38'31.2"E; **Event:** samplingProtocol: under the leaf litter; eventDate: 22.VI.2021; **Record Level:** basisOfRecord: PreservedSpecimen
- **Type status:** Other material. **Occurrence:** sex: 2 females; lifeStage: adult; **Taxon:** scientificName: Trichotichnus (Trichotichnus) miser; order: Coleoptera; family: Carabidae; genus: Trichotichnus; subgenus: Trichotichnus; specificEpithet: miser; scientificNameAuthorship: (Tschitschérine, 1897); vernacularName: 둥근가슴윤머리먼지벌레; **Location:** country: Korea; stateProvince: Daegu-si; locality: Suseong-gu, Hwanggeum-dong, Duribong, Coll. Dooyoung Kim; verbatimLatitude: 35°50'17.3"N; verbatimLongitude: 128°38'31.2"E; **Event:** samplingProtocol: under the leaf litter; eventDate: 30.VI.2021; **Record Level:** basisOfRecord: PreservedSpecimen
- **Type status:** Other material. **Occurrence:** sex: 2 males, 2 females; lifeStage: adult; **Taxon:** scientificName: Trichotichnus (Trichotichnus) miser; order: Coleoptera; family: Carabidae; genus: Trichotichnus; subgenus: Trichotichnus; specificEpithet: miser; scientificNameAuthorship: (Tschitschérine, 1897); vernacularName: 둥근가슴윤머리먼지벌레; **Location:** country: Korea; stateProvince: Gyeongsangnam-do; county: Geoje-si; locality: Irun-myeon, Wahyeon-ri, Is. Naedo, Coll. Dooyoung Kim, Donguk Kim; verbatimLatitude: 34°47'22.2"N; verbatimLongitude: 128°42'53.7"E; **Event:** samplingProtocol: under the leaf litter; eventDate: 12.XI.2021; **Record Level:** basisOfRecord: PreservedSpecimen

#### Description

Body length: 6.2–7.9 mm, width: 2.6–3.3 mm.

**Figure 4.**
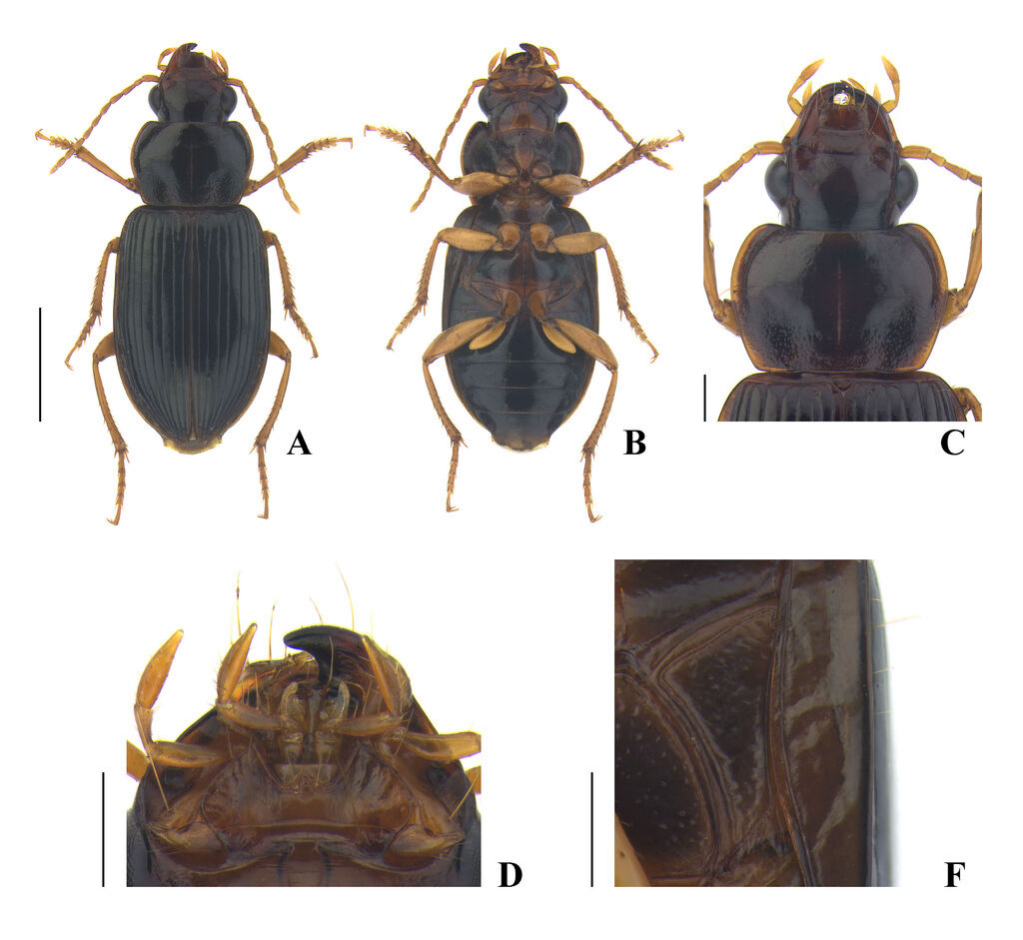
Trichotichnus (Trichotichnus) miser (Tschitschérine, 1897), male: **A** habitus, dorsal view; **B** ditto, ventral view; **C** head and pronotum, dorsal view; **D** head, ventral view; **E** metepisternum. Scale bars: 2 mm (**A, B**); 0.5 mm (**C**–**E**).

**Coloration** (Fig. 4A, B) shiny, black or reddish black, elytra somewhat iridescent; antennae, maxillary palpi, labial palpi, lateral margin of pronotum, and legs yellowish brown; labrum and mandibles dark reddish brown; apex of each mandible black; ventral side overall black, partially brownish.

**Head** (Fig. 4C, D) moderately convex, glabrous; eye prominent, distinctly convex; tempora very short and flat; mandible narrowly rounded at apex, not truncate; anterior margin of labrum moderately concave; anterior margin of clypeus slightly emarginate; frontal impression somewhat distinct, gently becoming shallow near eye; supraorbital seta located slightly before the level of hind margin of eye; microsculpture invisible on disc, consisting of faint transverse meshes near hind margin of eye and somewhat isodiametric meshes near supraorbital seta; postgena not ciliate; mentum and submentum fully separated by transverse suture; mentum with one distinct tooth, which is rounded at apex, epilobe slightly widened anteriorly, minutely projected beyond lateral lobe; submentum with a long seta on each side; ligula narrow on base, gently widened apically, apex truncate, with two apical setae; paraglossa somewhat narrow, separated from ligula by a quite wide notch; penultimate maxillary palpomere distinctly shorter than apical palpomere; penultimate labial palpomere slightly shorter than apical palpomere; antenna extending behind basal margin of pronotum.

**Pronotum** (Fig. 4C) moderately convex anteriorly, somewhat flat posteriorly, wider than long, widest slightly behind 1/3 from apex, PbW longer than PL; apical margin slightly or moderately emarginate, clearly bordered laterally, but faintly or not bordered in middle; apical angle rounded, protruding anteriorly; lateral margin without sinuation, almost linear posteriorly; lateral seta located slightly before widest point; basal margin almost straight, weakly bisinuate, slightly longer than apical margin, completely bordered, slightly interrupted in middle; basal angle distinctly obtuse, narrowly rounded, not protruding laterally; disc glabrous; median line clear, interrupted near base, not reaching apical border and reaching or not reaching basal border; anterior impression very faint; basal fovea shallow or slightly deep, gently curved outwards; basal area sparsely punctate in lateral and median area, rather distinctly punctate in basal fovea; lateral area sparsely punctate from base to the point near lateral seta and somewhat smooth or faintly punctate anteriorly; apical area rather variable in punctation, usually with a few faint punctures along apical margin, sometimes with more distinct punctures medially; microsculpture consisting of faint transverse meshes on disc. PW/HW = 1.37–1.44, mean 1.40; PW/PL = 1.44–1.55, mean 1.49; PW/PbW = 1.23–1.32, mean 1.27, in nine males and six females.

**Elytra** slightly convex, widest a little before middle; basal edge slightly curved, almost linear, forming an obtuse angle with lateral margin; humerus with minute acute tooth visible from behind; apical sinuation very weak; intervals overall flat, slightly convex near apex, without punctation; setigerous pore of interval 3 located near 3/5 from base; striae moderately deep; parascutellar striole moderately long, rarely reaching stria 1; marginal umbilicate series with 7–9 pores (usually 8 pores) in anterior group and 8–11 pores (usually 9–10 pores) in posterior group; microsculpture invisible on disc. Hind wing fully developed. EW/PW = 1.23–1.32, mean 1.27; EL/EW = 1.45–1.53, mean 1.49, in nine males and six females.

**Ventral side** (Fig. 4B, E) moderately convex; prosternum covered with very short and fine pubescence; median area of metasternum, median area of abdominal sternite III, and anteromedian area of IV more sparsely pubescence than prosternum; proepisternum sparsely and faintly punctate; meso-, metepisternum, and lateral area of metasternum sparsely punctate; metepisternum (Fig. 4E) distinctly longer than wide (ML/MW = 1.33–1.45, mean 1.38, in eight males and five females), anterior and inner margin bordered; apical margin of abdominal sternite VII widely rounded in male and somewhat narrowly rounded in female, with two pairs of setae in both sexes.

**Legs**. Protibia not longitudinally sulcate on dorsal side; metacoxa with two setae; metafemur with two setae in hind margin; metatarsus shorter than width of head (metatarsus/HW = 0.73–0.82, mean 0.78, in eight males and six females); metatarsomere 1 shorter than metatarsomere 2 and 3 combined, about 1.5 times longer than metatarsomere 2, as long as metatarsomere 5, but generally metatarsomere 5 slightly longer; tarsomere 5 usually with three setae, sometimes two setae in each ventrolateral margin.

**Figure 5.**
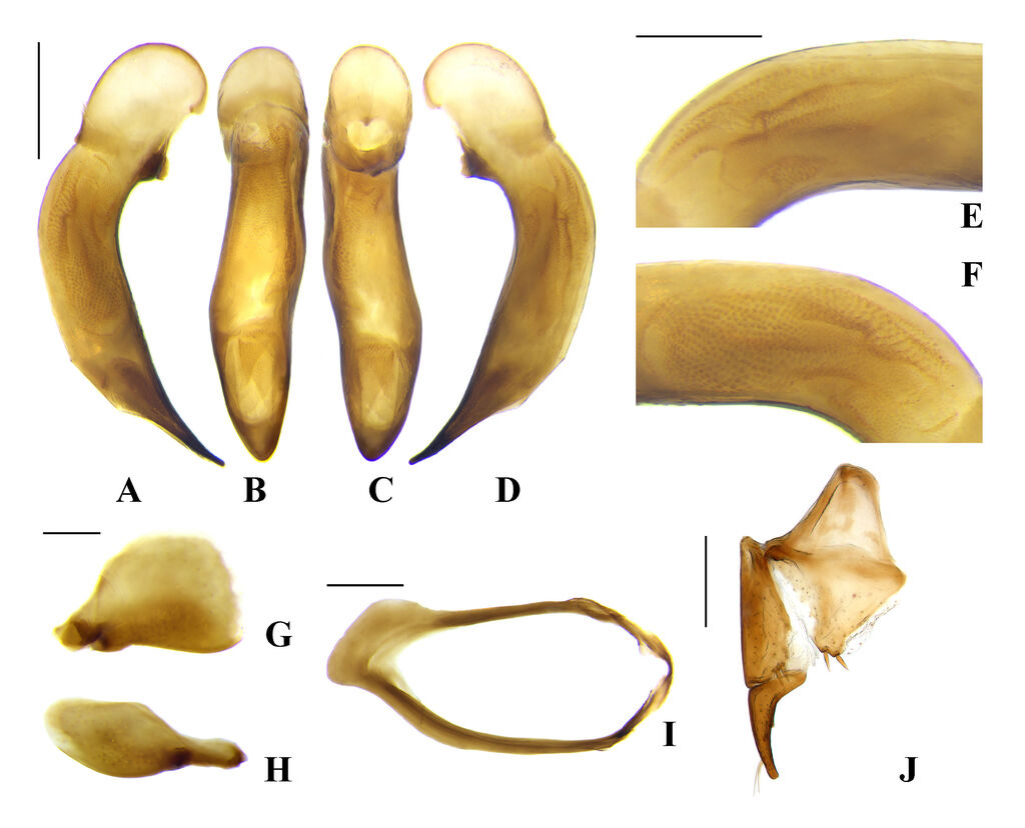
Male and female genitalia of Trichotichnus (Trichotichnus) miser (Tschitschérine, 1897): **A** median lobe of aedeagus, right lateral view; **B** ditto, dorsal view; **C** ditto, ventral view; **D** ditto, left lateral view; **E** internal sac of aedeagus, left lateral view; **F** ditto, right lateral view; **G** left paramere; **H** right paramere; **I** male sternite Ⅸ; **J** female genitalia. Scale bars: 0.5 mm (**A**–**D, I**); 0.3 mm (**E, F, J**); 0.2 mm (**G, H**).

**Male genitalia** (Fig. 5A–I). Median lobe of aedeagus (Fig. 5A–F) slender, moderately curved behind basal bulb and slightly curved before apex in lateral view, almost linear in dorsal view; terminal lamella slightly wider than long, gently contracted towards apex; apex somewhat narrowly rounded; internal sac covering right lateromedian part consisting of numerous round patches (Fig. 5F); internal sac without sclerotized part, but sometimes with a very small sclerotized piece; ventral side of median lobe bordered, rudimentarily serrate in middle; parameres as in Fig. 5G, H; sternite IX (Fig. 5I) explanate on basal area.

**Female genitalia** (Fig. 5J). Hemisternite with two spines apically; basal stylomere with one to three (usually two) preapical spines on external margin; apical stylomere slender, with two setae subapically, inner margin almost straight, slightly curved, outer margin gently curved, dorsal and ventral outer margin with one small distinct spine, respectively.

#### Diagnosis

The description of the *congruus*-group by Habu (1973) well matches with this species, except for the basal angle of the pronotum not being angulated. This species can be distinguished from others by pronotum with rounded basal angle, apical margin of abdominal sternite Ⅶ with two pairs of setae in both sexes, protibia not longitudinally sulcate on dorsal side, metatarsus shorter than width of head, and conformation of internal sac of aedeagus in right lateral view.

#### Distribution

Korea: South (new record), North (Kataev and Wrase 2017); China: Guizhou, Hubei, Hunan, Sichuan, Shaanxi, Zhejiang (Kataev and Wrase 2017).

#### Ecology

The immature adults were observed from June to July, whereas the mature adults were observed from late March to early May. Most individuals were observed under the leaf litter at the bottom of the drainage channel, located in the surroundings of the deciduous forest (Fig. 3A, B).

#### Notes

This species was first recorded in Korea by Kataev and Wrase (2017), based on the specimen from the northern part of Korea, and is recorded for the first time for the southern part of Korea by the current study. The specimens from the southern island of Geojedo Island (Naedo Island) might indicate the possibility of the occurrence of this species in Japan.

### Trichotichnus (Trichotichnus) vespertinus

Habu, 1954

A659A63C-1F7C-510F-8410-5390C6B1BDC5

Trichotichnus vespertinus Habu, 1954 - Habu 1954a: 57 (Japan: Mt. Hiko).
Trichotichnus habui Jedlička, 1958 - Jedlička 1958: 909 (Japan: Mt. Takao).
Trichotichnus (Trichotichnus) vespertinus : Habu, 1961 - Habu 1961: 145.

#### Materials

- **Type status:** Other material. **Occurrence:** sex: 1 male; lifeStage: adult; **Taxon:** scientificName: Trichotichnus (Trichotichnus) vespertinus; order: Coleoptera; family: Carabidae; genus: Trichotichnus; subgenus: Trichotichnus; specificEpithet: vespertinus; scientificNameAuthorship: Habu, 1954; vernacularName: 야산윤머리먼지벌레; **Location:** country: Korea; stateProvince: Daegu-si; locality: Suseong-gu, Beommul-dong, Jinbatgol, Coll. Dooyoung Kim; verbatimLatitude: 35°48'10.6"N; verbatimLongitude: 128°39'48.4"E; **Event:** samplingProtocol: under the leaf litter; eventDate: 26.IX.2018; **Record Level:** basisOfRecord: PreservedSpecimen
- **Type status:** Other material. **Occurrence:** sex: 1 male; lifeStage: adult; **Taxon:** scientificName: Trichotichnus (Trichotichnus) vespertinus; order: Coleoptera; family: Carabidae; genus: Trichotichnus; subgenus: Trichotichnus; specificEpithet: vespertinus; scientificNameAuthorship: Habu, 1954; vernacularName: 야산윤머리먼지벌레; **Location:** country: Korea; stateProvince: Daegu-si; locality: Suseong-gu, Jisan-dong, Joilgol, Coll. Dooyoung Kim; verbatimLatitude: 35°49'44.5"N; verbatimLongitude: 128°38'44.4"E; **Event:** samplingProtocol: attracted to light; eventDate: 3.IX.2018; **Record Level:** basisOfRecord: PreservedSpecimen
- **Type status:** Other material. **Occurrence:** sex: 1 female; lifeStage: adult; **Taxon:** scientificName: Trichotichnus (Trichotichnus) vespertinus; order: Coleoptera; family: Carabidae; genus: Trichotichnus; subgenus: Trichotichnus; specificEpithet: vespertinus; scientificNameAuthorship: Habu, 1954; vernacularName: 야산윤머리먼지벌레; **Location:** country: Korea; stateProvince: Daegu-si; locality: Suseong-gu, Jisan-dong, Joilgol, Coll. Dooyoung Kim; verbatimLatitude: 35°49'44.5"N; verbatimLongitude: 128°38'44.4"E; **Event:** samplingProtocol: attracted to light; eventDate: 5.IX.2018; **Record Level:** basisOfRecord: PreservedSpecimen
- **Type status:** Other material. **Occurrence:** sex: 1 female; lifeStage: adult; **Taxon:** scientificName: Trichotichnus (Trichotichnus) vespertinus; order: Coleoptera; family: Carabidae; genus: Trichotichnus; subgenus: Trichotichnus; specificEpithet: vespertinus; scientificNameAuthorship: Habu, 1954; vernacularName: 야산윤머리먼지벌레; **Location:** country: Korea; stateProvince: Daegu-si; locality: Suseong-gu, Hwanggeum-dong, Duribong, Coll. Dooyoung Kim; verbatimLatitude: 35°50'17.3"N; verbatimLongitude: 128°38'31.2"E; **Event:** samplingProtocol: under the leaf litter; eventDate: 31.Ⅴ.2018; **Record Level:** basisOfRecord: PreservedSpecimen
- **Type status:** Other material. **Occurrence:** sex: 2 females; lifeStage: adult; **Taxon:** scientificName: Trichotichnus (Trichotichnus) vespertinus; order: Coleoptera; family: Carabidae; genus: Trichotichnus; subgenus: Trichotichnus; specificEpithet: vespertinus; scientificNameAuthorship: Habu, 1954; vernacularName: 야산윤머리먼지벌레; **Location:** country: Korea; stateProvince: Daegu-si; locality: Suseong-gu, Hwanggeum-dong, Duribong, Coll. Dooyoung Kim; verbatimLatitude: 35°50'17.3"N; verbatimLongitude: 128°38'31.2"E; **Event:** samplingProtocol: under the leaf litter; eventDate: 6.VI.2018; **Record Level:** basisOfRecord: PreservedSpecimen
- **Type status:** Other material. **Occurrence:** sex: 1 male, 1 female; lifeStage: adult; **Taxon:** scientificName: Trichotichnus (Trichotichnus) vespertinus; order: Coleoptera; family: Carabidae; genus: Trichotichnus; subgenus: Trichotichnus; specificEpithet: vespertinus; scientificNameAuthorship: Habu, 1954; vernacularName: 야산윤머리먼지벌레; **Location:** country: Korea; stateProvince: Daegu-si; locality: Suseong-gu, Hwanggeum-dong, Duribong, Coll. Dooyoung Kim; verbatimLatitude: 35°50'17.3"N; verbatimLongitude: 128°38'31.2"E; **Event:** samplingProtocol: under the leaf litter; eventDate: 20.VI.2018; **Record Level:** basisOfRecord: PreservedSpecimen
- **Type status:** Other material. **Occurrence:** sex: 2 males, 1 female; lifeStage: adult; **Taxon:** scientificName: Trichotichnus (Trichotichnus) vespertinus; order: Coleoptera; family: Carabidae; genus: Trichotichnus; subgenus: Trichotichnus; specificEpithet: vespertinus; scientificNameAuthorship: Habu, 1954; vernacularName: 야산윤머리먼지벌레; **Location:** country: Korea; stateProvince: Daegu-si; locality: Suseong-gu, Hwanggeum-dong, Duribong, Coll. Dooyoung Kim; verbatimLatitude: 35°50'17.3"N; verbatimLongitude: 128°38'31.2"E; **Event:** samplingProtocol: under the leaf litter; eventDate: 13.VI.2019; **Record Level:** basisOfRecord: PreservedSpecimen
- **Type status:** Other material. **Occurrence:** sex: 1 male, 2 females; lifeStage: adult; **Taxon:** scientificName: Trichotichnus (Trichotichnus) vespertinus; order: Coleoptera; family: Carabidae; genus: Trichotichnus; subgenus: Trichotichnus; specificEpithet: vespertinus; scientificNameAuthorship: Habu, 1954; vernacularName: 야산윤머리먼지벌레; **Location:** country: Korea; stateProvince: Daegu-si; locality: Suseong-gu, Hwanggeum-dong, Duribong, Coll. Dooyoung Kim; verbatimLatitude: 35°50'17.3"N; verbatimLongitude: 128°38'31.2"E; **Event:** samplingProtocol: under the leaf litter; eventDate: 18.VI.2019; **Record Level:** basisOfRecord: PreservedSpecimen
- **Type status:** Other material. **Occurrence:** sex: 1 female; lifeStage: adult; **Taxon:** scientificName: Trichotichnus (Trichotichnus) vespertinus; order: Coleoptera; family: Carabidae; genus: Trichotichnus; subgenus: Trichotichnus; specificEpithet: vespertinus; scientificNameAuthorship: Habu, 1954; vernacularName: 야산윤머리먼지벌레; **Location:** country: Korea; stateProvince: Daegu-si; locality: Suseong-gu, Hwanggeum-dong, Duribong, Coll. Dooyoung Kim; verbatimLatitude: 35°50'17.3"N; verbatimLongitude: 128°38'31.2"E; **Event:** samplingProtocol: under the leaf litter; eventDate: 23.VI.2019; **Record Level:** basisOfRecord: PreservedSpecimen
- **Type status:** Other material. **Occurrence:** sex: 1 female; lifeStage: adult; **Taxon:** scientificName: Trichotichnus (Trichotichnus) vespertinus; order: Coleoptera; family: Carabidae; genus: Trichotichnus; subgenus: Trichotichnus; specificEpithet: vespertinus; scientificNameAuthorship: Habu, 1954; vernacularName: 야산윤머리먼지벌레; **Location:** country: Korea; stateProvince: Daegu-si; locality: Suseong-gu, Hwanggeum-dong, Duribong, Coll. Dooyoung Kim; verbatimLatitude: 35°50'17.3"N; verbatimLongitude: 128°38'31.2"E; **Event:** samplingProtocol: under the leaf litter; eventDate: 4.VII.2019; **Record Level:** basisOfRecord: PreservedSpecimen
- **Type status:** Other material. **Occurrence:** sex: 1 male; lifeStage: adult; **Taxon:** scientificName: Trichotichnus (Trichotichnus) vespertinus; order: Coleoptera; family: Carabidae; genus: Trichotichnus; subgenus: Trichotichnus; specificEpithet: vespertinus; scientificNameAuthorship: Habu, 1954; vernacularName: 야산윤머리먼지벌레; **Location:** country: Korea; stateProvince: Daegu-si; locality: Suseong-gu, Hwanggeum-dong, Duribong, Coll. Dooyoung Kim; verbatimLatitude: 35°50'17.3"N; verbatimLongitude: 128°38'31.2"E; **Event:** samplingProtocol: under the leaf litter; eventDate: 7.VII.2019; **Record Level:** basisOfRecord: PreservedSpecimen
- **Type status:** Other material. **Occurrence:** sex: 1 female; lifeStage: adult; **Taxon:** scientificName: Trichotichnus (Trichotichnus) vespertinus; order: Coleoptera; family: Carabidae; genus: Trichotichnus; subgenus: Trichotichnus; specificEpithet: vespertinus; scientificNameAuthorship: Habu, 1954; vernacularName: 야산윤머리먼지벌레; **Location:** country: Korea; stateProvince: Daegu-si; locality: Suseong-gu, Hwanggeum-dong, Duribong, Coll. Dooyoung Kim; verbatimLatitude: 35°50'17.3"N; verbatimLongitude: 128°38'31.2"E; **Event:** samplingProtocol: under the leaf litter; eventDate: 14.Ⅴ.2021; **Record Level:** basisOfRecord: PreservedSpecimen
- **Type status:** Other material. **Occurrence:** sex: 1 male; lifeStage: adult; **Taxon:** scientificName: Trichotichnus (Trichotichnus) vespertinus; order: Coleoptera; family: Carabidae; genus: Trichotichnus; subgenus: Trichotichnus; specificEpithet: vespertinus; scientificNameAuthorship: Habu, 1954; vernacularName: 야산윤머리먼지벌레; **Location:** country: Korea; stateProvince: Daegu-si; locality: Suseong-gu, Hwanggeum-dong, Duribong, Coll. Dooyoung Kim; verbatimLatitude: 35°50'17.3"N; verbatimLongitude: 128°38'31.2"E; **Event:** samplingProtocol: under the leaf litter; eventDate: 19.Ⅴ.2021; **Record Level:** basisOfRecord: PreservedSpecimen
- **Type status:** Other material. **Occurrence:** sex: 1 female; lifeStage: adult; **Taxon:** scientificName: Trichotichnus (Trichotichnus) vespertinus; order: Coleoptera; family: Carabidae; genus: Trichotichnus; subgenus: Trichotichnus; specificEpithet: vespertinus; scientificNameAuthorship: Habu, 1954; vernacularName: 야산윤머리먼지벌레; **Location:** country: Korea; stateProvince: Daegu-si; locality: Suseong-gu, Hwanggeum-dong, Duribong, Coll. Dooyoung Kim; verbatimLatitude: 35°50'17.3"N; verbatimLongitude: 128°38'31.2"E; **Event:** samplingProtocol: under the leaf litter; eventDate: 25.Ⅴ.2021; **Record Level:** basisOfRecord: PreservedSpecimen
- **Type status:** Other material. **Occurrence:** sex: 1 female; lifeStage: adult; **Taxon:** scientificName: Trichotichnus (Trichotichnus) vespertinus; order: Coleoptera; family: Carabidae; genus: Trichotichnus; subgenus: Trichotichnus; specificEpithet: vespertinus; scientificNameAuthorship: Habu, 1954; vernacularName: 야산윤머리먼지벌레; **Location:** country: Korea; stateProvince: Daegu-si; locality: Suseong-gu, Hwanggeum-dong, Duribong, Coll. Dooyoung Kim; verbatimLatitude: 35°50'17.3"N; verbatimLongitude: 128°38'31.2"E; **Event:** samplingProtocol: under the leaf litter; eventDate: 29.Ⅴ.2021; **Record Level:** basisOfRecord: PreservedSpecimen
- **Type status:** Other material. **Occurrence:** sex: 5 males, 3 females; lifeStage: adult; **Taxon:** scientificName: Trichotichnus (Trichotichnus) vespertinus; order: Coleoptera; family: Carabidae; genus: Trichotichnus; subgenus: Trichotichnus; specificEpithet: vespertinus; scientificNameAuthorship: Habu, 1954; vernacularName: 야산윤머리먼지벌레; **Location:** country: Korea; stateProvince: Daegu-si; locality: Suseong-gu, Hwanggeum-dong, Duribong, Coll. Dooyoung Kim; verbatimLatitude: 35°50'17.3"N; verbatimLongitude: 128°38'31.2"E; **Event:** samplingProtocol: under the leaf litter; eventDate: 6.Ⅹ.2021; **Record Level:** basisOfRecord: PreservedSpecimen
- **Type status:** Other material. **Occurrence:** sex: 1 male; lifeStage: adult; **Taxon:** scientificName: Trichotichnus (Trichotichnus) vespertinus; order: Coleoptera; family: Carabidae; genus: Trichotichnus; subgenus: Trichotichnus; specificEpithet: vespertinus; scientificNameAuthorship: Habu, 1954; vernacularName: 야산윤머리먼지벌레; **Location:** country: Korea; stateProvince: Daegu-si; locality: Suseong-gu, Hwanggeum-dong, Mt. Muhaksan, Coll. Dooyoung Kim; verbatimLatitude: 35°50'13.9"N; verbatimLongitude: 128°38'19.1"E; **Event:** samplingProtocol: under the leaf litter; eventDate: 5.VI.2021; **Record Level:** basisOfRecord: PreservedSpecimen
- **Type status:** Other material. **Occurrence:** sex: 1 male; lifeStage: adult; **Taxon:** scientificName: Trichotichnus (Trichotichnus) vespertinus; order: Coleoptera; family: Carabidae; genus: Trichotichnus; subgenus: Trichotichnus; specificEpithet: vespertinus; scientificNameAuthorship: Habu, 1954; vernacularName: 야산윤머리먼지벌레; **Location:** country: Korea; stateProvince: Daegu-si; locality: Suseong-gu, Hwanggeum-dong, Mt. Muhaksan, Coll. Dooyoung Kim; verbatimLatitude: 35°50'13.9"N; verbatimLongitude: 128°38'19.1"E; **Event:** samplingProtocol: under the leaf litter; eventDate: 6.X.2021; **Record Level:** basisOfRecord: PreservedSpecimen
- **Type status:** Other material. **Occurrence:** sex: 1 male; lifeStage: adult; **Taxon:** scientificName: Trichotichnus (Trichotichnus) vespertinus; order: Coleoptera; family: Carabidae; genus: Trichotichnus; subgenus: Trichotichnus; specificEpithet: vespertinus; scientificNameAuthorship: Habu, 1954; vernacularName: 야산윤머리먼지벌레; **Location:** country: Korea; stateProvince: Daegu-si; locality: Suseong-gu, Hwanggeum-dong, Beomo Park, Coll. Dooyoung Kim; verbatimLatitude: 35°50'47.6"N; verbatimLongitude: 128°38'08.4"E; **Event:** samplingProtocol: under the leaf litter; eventDate: 6. IX.2018; **Record Level:** basisOfRecord: PreservedSpecimen

#### Description

Body length: 9.5–10.3 mm, width: 3.9–4.1 mm.

**Figure 6.**
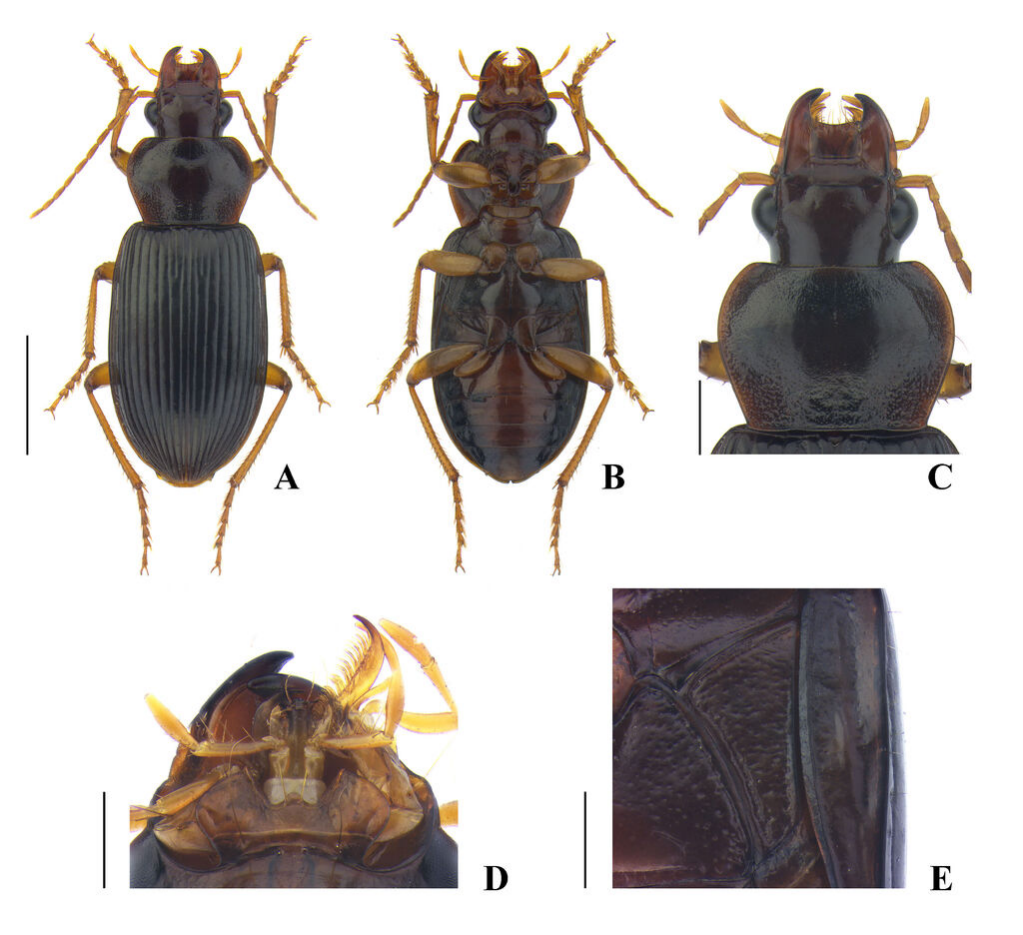
Trichotichnus (Trichotichnus) vespertinus Habu, 1954, male: **A** habitus, dorsal view; **B** ditto, ventral view; **C** head and pronotum, dorsal view; **D** head, ventral view; **E** metepisternum. Scale bars: 3 mm (**A, B**); 1 mm (**C**); 0.6 mm (**D, E**).

**Coloration** (Fig. 6A, B) shiny, black or reddish black, elytra iridescent; antennae, maxillary palpi, labial palpi, and legs yellowish brown; labrum, mandibles, and lateral margin of pronotum reddish brown; apex of each mandible black; ventral side overall black, partially brownish.

**Head** (Fig. 6C, D) moderately convex, glabrous; eye prominent, distinctly convex; tempora flat, weakly developed; mandible narrowly rounded at apex, not truncate; anterior margin of labrum gently concave; anterior margin of clypeus almost linear; frontal impression somewhat distinct, gently becoming shallow near eye; supraorbital seta located before the level of hind margin of eye; microsculpture invisible on disc, consisting of faint transverse meshes near supraorbital seta; postgena not ciliate; mentum and submentum fully separated by transverse suture; mentum with one stout tooth, which is widely rounded at apex, epilobe narrow, usually not widened anteriorly, minutely projected beyond lateral lobe; submentum with a pair of long seta; ligula wide, drastically widened at apex, apex truncate, with two apical setae; paraglossa distinctly narrower than ligula, separated from ligula by wide notch apically; penultimate maxillary palpomere slightly longer than apical palpomere; penultimate labial palpomere distinctly longer than apical palpomere; antenna extending behind basal margin of pronotum.

**Pronotum** (Fig. 6C) moderately convex anteriorly, somewhat flat posteriorly, wider than long, widest slightly before 2/5 from apex, PbW longer than PL; apical margin slightly emarginate, completely bordered laterally, faint in middle; apical angle rounded, weakly protruding anteriorly; lateral margin slightly sinuate before base; lateral seta located near widest point; basal margin almost straight, slightly bisinuate, longer than apical margin, completely bordered; basal angle obtuse, angulate, slightly protruding laterally; median line shallow, rather clear in middle, interrupted near apex and base; anterior impression quite distinct; basal fovea shallow, slightly curved outwards; surface densely punctate, except on disc, which is faintly and sparsely punctate; microsculpture invisible on disc. PW/HW = 1.40–1.44, mean 1.42; PW/PL = 1.44–1.50, mean 1.47; PW/PbW = 1.29–1.36, mean 1.31, in four males and three females.

**Elytra** moderately convex, widest before middle; basal edge weakly sinuate medially, forming an obtuse angle with lateral margin; humerus with minute acute tooth visible from behind; apical sinuation very weak; intervals somewhat flat, slightly convex near lateral area and apex, without punctation; setigerous pore of interval 3 located a little behind middle; striae somewhat deep; parascutellar striole moderately long, usually not reaching stria 1; marginal umbilicate series with 21–24 pores; microsculpture invisible on disc. Hind wing fully developed. EW/PW = 1.25–1.32, mean 1.28; EL/EW = 1.55–1.63, mean 1.59, in four males and three females.

**Ventral side** (Fig. 6B, E) moderately convex; prosternum covered with very short and fine pubescence; median area of metasternum, median area of abdominal sternite III, and anteromedian area of abdominal sternite IV more sparsely pubescence than prosternum; proepisternum vaguely punctate; meso-, metepisternum, and lateral area of metasternum moderately punctate; metepisternum (Fig. 6E) distinctly longer than wide (ML/MW = 1.43–1.52, mean 1.48, in three males and three females); anterior and inner margin bordered; apical margin of abdominal sternite VII widely rounded in both sexes, with one pair of setae in male and two pairs of setae in female.

**Legs**. Protibia longitudinally sulcate on dorsal side; metacoxa with two setae; metafemur with two setae in hind margin; metatarsomere 1 distinctly shorter than metatarsomere 2 and 3 combined, slightly shorter than metatarsomere 5; pro- and mesotarsomere 5 with three setae in each ventrolateral margin and metatarsomere 5 with four setae in each ventrolateral margin.

**Figure 7.**
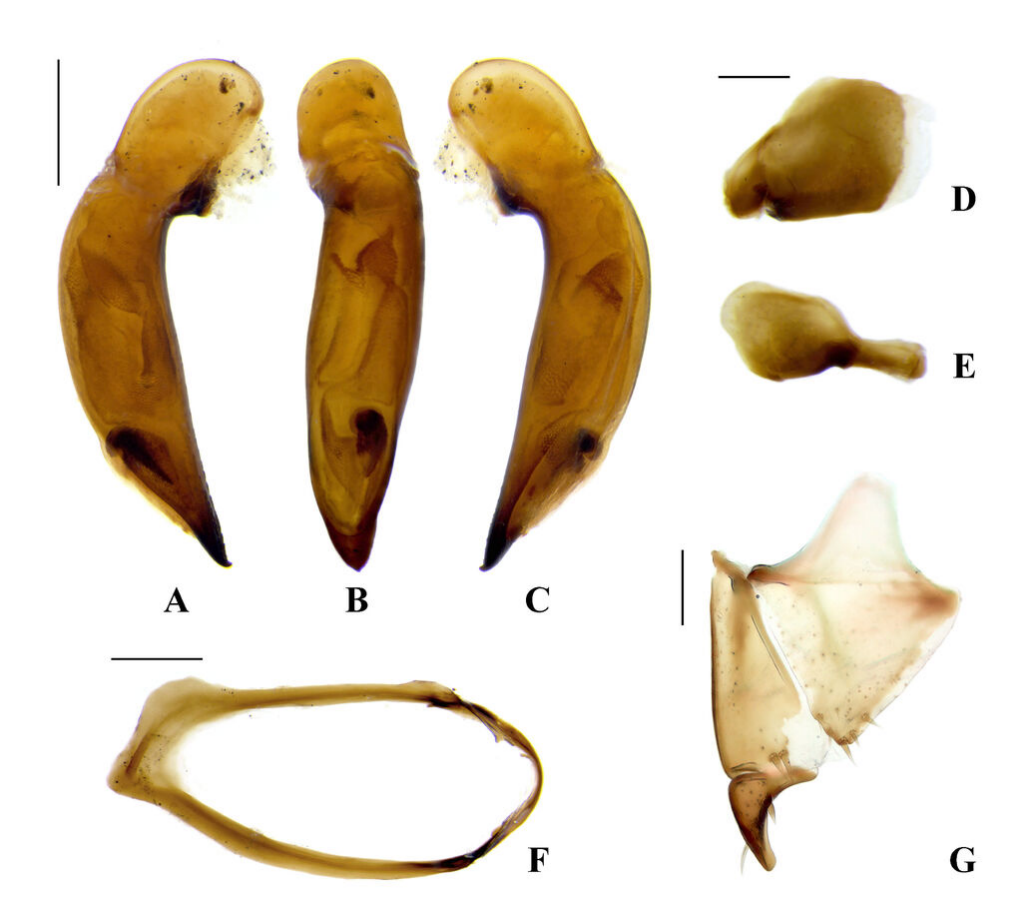
Male and female genitalia of Trichotichnus (Trichotichnus) vespertinus Habu, 1954: **A** median lobe of aedeagus, right lateral view; **B** ditto, dorsal view; **C** ditto, left lateral view; **D** left paramere; **E** right paramere; **F** male sternite Ⅸ; **G** female genitalia. Scale bars: 0.6 mm (**A**–**C, F**); 0.3 mm (**D, E, G**).

**Male genitalia** (Fig. 7A–F). Median lobe of aedeagus (Fig. 7A–C) gently curved on dorsal side and almost linear between basal bulb and apex on ventral side in lateral view, almost linear, slightly curved to left side in dorsal view; terminal lamella wider than long; apex minutely hooked ventrally, which looks minutely pointed in dorsal view; large sclerotized spiny hook present at subapical area; ventral side of median lobe bordered with two parallel serrate lines; parameres as in Fig. 7D and E; sternite IX (Fig. 7F) explanate on basal area.

**Female genitalia** (Fig. 7G). Hemisternite with two to three spines (rarely more than three) apically; basal stylomere with two preapical spines on external margin; apical stylomere short and stout, truncate at apex, with two setae subapically, inner margin slightly curved, outer margin well curved throughout, dorsal and ventral outer margin with one distinct spine, respectively.

#### Diagnosis

This species can be easily identified by the following significant features of the male and female genitalia: median lobe of aedeagus in apical part minutely hooked ventrally, ventral side bordered with two parallel serrate lines, internal sac with distinct sclerotized spiny hook in subapical area; apical stylomere short and stout.

#### Distribution

Korea: South (new record); Japan: Honshu, Shikoku, Kyushu (Habu 1973).

#### Ecology

The immature adults were observed from mid-May to June, the mature adults were observed from September to early October and the mating behavior was observed in early October. Most individuals were observed under the leaf litter at the bottom of the drainage channel, located in the surroundings of the deciduous forest (Fig. 3A, D) and some were attracted to light. According to Habu (1954c), this species was usually collected in the low mountain areas and frequently attracted to light.

#### Notes

This species was previously only known to occur in Japan, but a new distribution record is added to Korea.

### Trichotichnus (Iridessus) lucidus

(Morawitz, 1863)

B5FCA821-39BD-5409-8EC9-9A6D4A50F5FE

Harpalus lucidus Morawitz, 1863 - Morawitz 1863: 72.
Iridessus lucidus : Bates, 1883 - Bates 1883: 240.
Trichotichnus lucidus : Csiki, 1932 - Csiki 1932: 1219.
Trichotichnus (Trichotichnus) lucidus : Habu, 1961 - Habu 1961: 135.
Trichotichnus (Iridessus) lucidus : Kataev and Wrase, 2017 - *Kataev and Wrase 2017*: 558.

#### Materials

- **Type status:** Other material. **Occurrence:** sex: 1 male; lifeStage: adult; **Taxon:** scientificName: Trichotichnus (Iridessus) lucidus; order: Coleoptera; family: Carabidae; genus: Trichotichnus; subgenus: Iridessus; specificEpithet: lucidus; scientificNameAuthorship: (Morawitz, 1863); vernacularName: 참윤머리먼지벌레; **Location:** country: Korea; stateProvince: Jeju-si; locality: Mt. Hallasan, Coll. Tae Sung Kwon; **Event:** samplingProtocol: pit-fall trap; eventTime: V–VI.2011; **Record Level:** basisOfRecord: PreservedSpecimen

#### Distribution

Korea: South; Japan: Kyushu, Honshu, Shikoku, Hokkaido (Habu 1973); Russia: Far East (Kataev and Wrase 2017).

#### Notes

**Figure 8.**
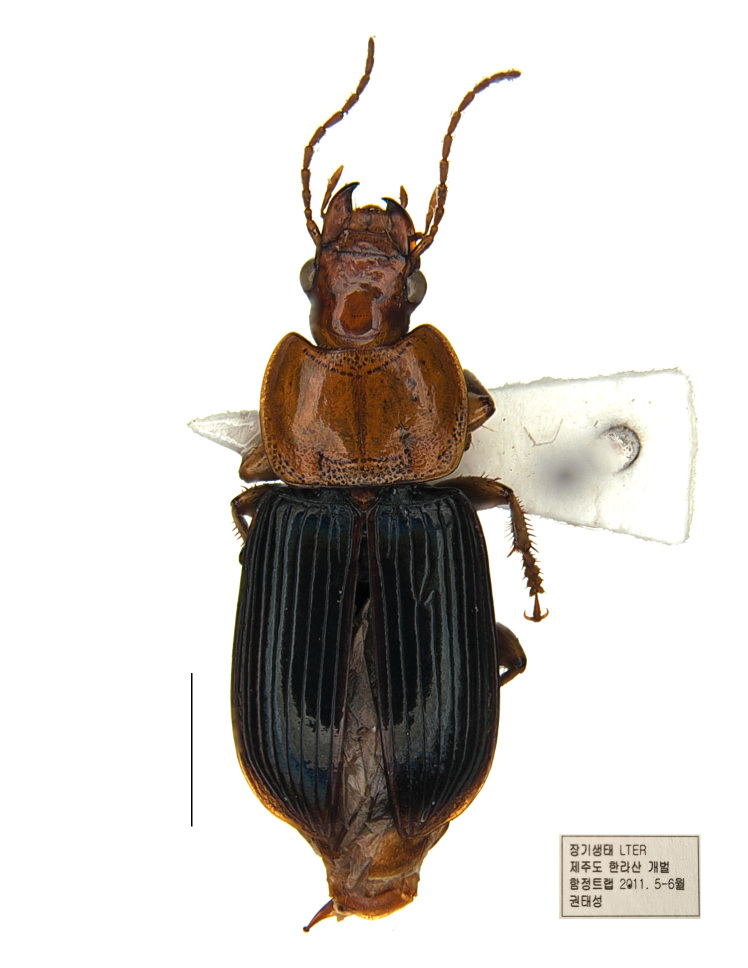
Habitus of Trichotichnus (Iridessus) lucidus (Morawitz, 1863), male. Scale bar: 2 mm.

Moon and Paik (2006c) mentioned that the Korean specimen of *T.lucidus* was not identified during the study. However, the brief collection data and photography of this species in Korea was recently recorded by Kwon et al. (2018), based on the specimen deposited in the National Institute of Forest Science, Korea. The specimen was re-examined by the authors and the distribution of *T.lucidus* in Korea is confirmed (Fig. 8).

### Trichotichnus (Trichotichnus) longitarsis

Morawitz, 1863

CA5CFC06-9B3F-5AA4-ADE9-18246BCB1619

Trichotichnus longitarsis Morawitz, 1863 - Morawitz 1863: 65.
Trichotichnus (Trichotichnus) longitarsis : Habu, 1961 - Habu 1961: 146.

#### Materials

- **Type status:** Other material. **Occurrence:** sex: 32 males, 12 females; lifeStage: adult; **Taxon:** scientificName: Trichotichnus (Trichotichnus) longitarsis; order: Coleoptera; family: Carabidae; genus: Trichotichnus; subgenus: Trichotichnus; specificEpithet: longitarsis; scientificNameAuthorship: Morawitz, 1863; vernacularName: 붉은윤머리먼지벌레; **Location:** country: Korea; stateProvince: Daegu-si; locality: Dalseong-gun, Gachang-myeon, Ju-ri, Mt. Choejeongsan, Coll. Dooyoung Kim; verbatimLatitude: 35°44'47.8"N; verbatimLongitude: 128°36'20.1"E; **Event:** samplingProtocol: under the leaf litter; eventDate: 19.IX.2021; **Record Level:** basisOfRecord: PreservedSpecimen
- **Type status:** Other material. **Occurrence:** sex: 1 female; lifeStage: adult; **Taxon:** scientificName: Trichotichnus (Trichotichnus) longitarsis; order: Coleoptera; family: Carabidae; genus: Trichotichnus; subgenus: Trichotichnus; specificEpithet: longitarsis; scientificNameAuthorship: Morawitz, 1863; vernacularName: 붉은윤머리먼지벌레; **Location:** country: Korea; stateProvince: Daegu-si; locality: Suseong-gu, Beommul-dong, Jinbatgol, Coll. Dooyoung Kim; verbatimLatitude: 35°47'55.3"N; verbatimLongitude: 128°40'00.9"E; **Event:** samplingProtocol: searching on ground surface; eventDate: 5.X.2020; **Record Level:** basisOfRecord: PreservedSpecimen
- **Type status:** Other material. **Occurrence:** sex: 1 male; lifeStage: adult; **Taxon:** scientificName: Trichotichnus (Trichotichnus) longitarsis; order: Coleoptera; family: Carabidae; genus: Trichotichnus; subgenus: Trichotichnus; specificEpithet: longitarsis; scientificNameAuthorship: Morawitz, 1863; vernacularName: 붉은윤머리먼지벌레; **Location:** country: Korea; stateProvince: Daegu-si; locality: Suseong-gu, Jisan-dong, Joilgol, Coll. Dooyoung Kim; verbatimLatitude: 35°49'44.9"N; verbatimLongitude: 128°38'46.5"E; **Event:** samplingProtocol: attracted to light; eventDate: 28.VIII.2018; **Record Level:** basisOfRecord: PreservedSpecimen
- **Type status:** Other material. **Occurrence:** sex: 1 male; lifeStage: adult; **Taxon:** scientificName: Trichotichnus (Trichotichnus) longitarsis; order: Coleoptera; family: Carabidae; genus: Trichotichnus; subgenus: Trichotichnus; specificEpithet: longitarsis; scientificNameAuthorship: Morawitz, 1863; vernacularName: 붉은윤머리먼지벌레; **Location:** country: Korea; stateProvince: Daegu-si; locality: Suseong-gu, Hwanggeum-dong, Duribong, Coll. Dooyoung Kim; verbatimLatitude: 35°50'16.5"N; verbatimLongitude: 128°38'29.1"E; **Event:** samplingProtocol: under the leaf litter; eventDate: 15.V.2018; **Record Level:** basisOfRecord: PreservedSpecimen
- **Type status:** Other material. **Occurrence:** sex: 1 female; lifeStage: adult; **Taxon:** scientificName: Trichotichnus (Trichotichnus) longitarsis; order: Coleoptera; family: Carabidae; genus: Trichotichnus; subgenus: Trichotichnus; specificEpithet: longitarsis; scientificNameAuthorship: Morawitz, 1863; vernacularName: 붉은윤머리먼지벌레; **Location:** country: Korea; stateProvince: Gyeongsangbuk-do; locality: Gunwi-gun, Bugye-eup, Namsan-ri, Coll. Dooyoung Kim; verbatimLatitude: 36°01'23.0"N; verbatimLongitude: 128°37'54.4"E; **Event:** samplingProtocol: by digging out the soil; eventDate: 28.XII.2020; **Record Level:** basisOfRecord: PreservedSpecimen

#### Distribution

Korea: South; Japan: Kyushu, Honshu, Shikoku, Hokkaido (Habu 1973); Russia: Far East (Kataev and Wrase 2017).

### Trichotichnus (Trichotichnus) nishioi

Habu, 1961

0AC0B2B6-4109-5198-9BB9-4FF02B4C4CF4

Trichotichnus (Trichotichnus) nishioi Habu, 1961 - Habu 1961: 141.

#### Materials

- **Type status:** Other material. **Occurrence:** sex: 2 males, 1 female; lifeStage: adult; **Taxon:** scientificName: Trichotichnus (Trichotichnus) nishioi; order: Coleoptera; family: Carabidae; genus: Trichotichnus; subgenus: Trichotichnus; specificEpithet: nishioi; scientificNameAuthorship: Habu, 1961; vernacularName: 백운윤머리먼지벌레; **Location:** country: Korea; stateProvince: Daegu-si; locality: Dalseong-gun, Gachang-myeon, Ju-ri, Mt. Choejeongsan, Coll. Dooyoung Kim; verbatimLatitude: 35°44'47.8"N; verbatimLongitude: 128°36'20.1"E; **Event:** samplingProtocol: under the leaf litter; eventDate: 31.VII.2021; **Record Level:** basisOfRecord: PreservedSpecimen
- **Type status:** Other material. **Occurrence:** sex: 1 male, 1 female; lifeStage: adult; **Taxon:** scientificName: Trichotichnus (Trichotichnus) nishioi; order: Coleoptera; family: Carabidae; genus: Trichotichnus; subgenus: Trichotichnus; specificEpithet: nishioi; scientificNameAuthorship: Habu, 1961; vernacularName: 백운윤머리먼지벌레; **Location:** country: Korea; stateProvince: Daegu-si; locality: Dalseong-gun, Gachang-myeon, Ju-ri, Mt. Choejeongsan, Coll. Dooyoung Kim; verbatimLatitude: 35°44'47.8"N; verbatimLongitude: 128°36'20.1"E; **Event:** samplingProtocol: under the leaf litter; eventDate: 19.IX.2021; **Record Level:** basisOfRecord: PreservedSpecimen
- **Type status:** Other material. **Occurrence:** sex: 8 males, 6 females; lifeStage: adult; **Taxon:** scientificName: Trichotichnus (Trichotichnus) nishioi; order: Coleoptera; family: Carabidae; genus: Trichotichnus; subgenus: Trichotichnus; specificEpithet: nishioi; scientificNameAuthorship: Habu, 1961; vernacularName: 백운윤머리먼지벌레; **Location:** country: Korea; stateProvince: Daegu-si; locality: Dalseong-gun, Gachang-myeon, Ju-ri, Mt. Choejeongsan, Coll. Dooyoung Kim; verbatimLatitude: 35°44'47.8"N; verbatimLongitude: 128°36'20.1"E; **Event:** samplingProtocol: under the leaf litter; eventDate: 2.IV.2022; **Record Level:** basisOfRecord: PreservedSpecimen
- **Type status:** Other material. **Occurrence:** sex: 2 males; lifeStage: adult; **Taxon:** scientificName: Trichotichnus (Trichotichnus) nishioi; order: Coleoptera; family: Carabidae; genus: Trichotichnus; subgenus: Trichotichnus; specificEpithet: nishioi; scientificNameAuthorship: Habu, 1961; vernacularName: 백운윤머리먼지벌레; **Location:** country: Korea; stateProvince: Daegu-si; locality: Suseong-gu, Beommul-dong, Jinbatgol, Coll. Dooyoung Kim; verbatimLatitude: 35°48'36.5"N; verbatimLongitude: 128°39'45.1"E; **Event:** samplingProtocol: under the leaf litter; eventDate: 26.IX.2018; **Record Level:** basisOfRecord: PreservedSpecimen
- **Type status:** Other material. **Occurrence:** sex: 2 males; lifeStage: adult; **Taxon:** scientificName: Trichotichnus (Trichotichnus) nishioi; order: Coleoptera; family: Carabidae; genus: Trichotichnus; subgenus: Trichotichnus; specificEpithet: nishioi; scientificNameAuthorship: Habu, 1961; vernacularName: 백운윤머리먼지벌레; **Location:** country: Korea; stateProvince: Daegu-si; locality: Suseong-gu, Hwanggeum-dong, Duribong, Coll. Dooyoung Kim; verbatimLatitude: 35°50'17.3"N; verbatimLongitude: 128°38'31.2"E; **Event:** samplingProtocol: under the leaf litter; eventDate: 15.V.2018; **Record Level:** basisOfRecord: PreservedSpecimen
- **Type status:** Other material. **Occurrence:** sex: 2 males, 1 female; lifeStage: adult; **Taxon:** scientificName: Trichotichnus (Trichotichnus) nishioi; order: Coleoptera; family: Carabidae; genus: Trichotichnus; subgenus: Trichotichnus; specificEpithet: nishioi; scientificNameAuthorship: Habu, 1961; vernacularName: 백운윤머리먼지벌레; **Location:** country: Korea; stateProvince: Gyeongsangbuk-do; county: Yeongju-si; locality: Bonghyeon-myeon, Dusan-ri, Coll. Dooyoung Kim; verbatimLatitude: 36°50'39.0"N; verbatimLongitude: 128°28'02.6"E; **Event:** samplingProtocol: under the leaf litter; eventDate: 29.VII.2021; **Record Level:** basisOfRecord: PreservedSpecimen
- **Type status:** Other material. **Occurrence:** sex: 1 male; lifeStage: adult; **Taxon:** scientificName: Trichotichnus (Trichotichnus) nishioi; order: Coleoptera; family: Carabidae; genus: Trichotichnus; subgenus: Trichotichnus; specificEpithet: nishioi; scientificNameAuthorship: Habu, 1961; vernacularName: 백운윤머리먼지벌레; **Location:** country: Korea; stateProvince: Gyeongsangnam-do; locality: Sancheong-gun, Sicheon-myeon, Dongdang-ri, Mt. Gugoksan, Coll. Dooyoung Kim; verbatimLatitude: 35°17'31.8"N; verbatimLongitude: 127°47'16.0"E; **Event:** samplingProtocol: under the leaf litter; eventDate: 15.VIII.2021; **Record Level:** basisOfRecord: PreservedSpecimen

#### Distribution

Korea: South, North (Kataev and Wrase 2017); China: Beijing, Hebei (Kataev and Wrase 2017); Japan: Honshu, Hokkaido (Habu 1973); Russia: Far East (Kataev and Wrase 2017).

#### Ecology

The immature adults were observed around July and the mature adults were observed around April and October.

### Trichotichnus (Trichotichnus) noctuabundus

Habu, 1954

1F24589F-C1D9-524C-804B-9529C527670C

Trichotichnus noctuabundus Habu, 1954 - Habu 1954a: 56.
Trichotichnus (Trichotichnus) noctuabundus : Habu, 1961 - Habu 1961: 145.

#### Materials

- **Type status:** Other material. **Occurrence:** sex: 2 males, 2 females; lifeStage: adult; **Taxon:** scientificName: Trichotichnus (Trichotichnus) noctuabundus; order: Coleoptera; family: Carabidae; genus: Trichotichnus; subgenus: Trichotichnus; specificEpithet: noctuabundus; scientificNameAuthorship: Habu, 1954; vernacularName: 섬윤머리먼지벌레; **Location:** country: Korea; stateProvince: Gyeongsangnam-do; county: Geoje-si; locality: Irun-myeon, Wahyeon-ri, Is. Naedo, Coll. Dooyoung Kim, Donguk Kim; verbatimLatitude: 34°47'22.2"N; verbatimLongitude: 128°42'53.7"E; **Event:** samplingProtocol: under the leaf litter; eventDate: 12.XI.2021; **Record Level:** basisOfRecord: PreservedSpecimen

#### Distribution

Korea: South; China: Fujian, Hubei, Sichuan, Zhejiang, Taiwan (Kataev and Wrase 2017); Japan: Kyushu, Honshu (Habu 1973).

#### Notes

In Korea, this species has been only recorded from Jejudo Island, but it was also collected from the southern island of Geojedo Island by the authors.

## Checklists

### Updated checklist of *Trichotichnus* species known to occur in Korea.

#### Trichotichnus (Bottchrus) nanus spinulosus

Habu, 1954

D4589351-04DC-525C-8360-E503EBDB92C0

#### Trichotichnus (Iridessus) lucidus

(Morawitz, 1863)

EAEB7952-B76C-5634-BE1F-8EFBB8872833

#### Trichotichnus (Trichotichnus) congruus

Motschulsky, 1866

6669F7CD-DFFB-5267-B2D8-DB07E2FD10B2

#### Trichotichnus (Trichotichnus) coruscus coruscus

(Tschitschérine, 1895)

4979C6EB-B1B5-504B-9781-0EB48BF9BAF7

#### Trichotichnus (Trichotichnus) longitarsis

Morawitz, 1863

071C421B-9543-54A9-B997-D73C6E29EB3D

#### Trichotichnus (Trichotichnus) miser

(Tschitschérine, 1897)

67EA2A7E-AC6C-53A8-8099-7504B0E358AB

#### Trichotichnus (Trichotichnus) nishioi

Habu, 1961

E0E0D60A-0864-51D3-B263-8ED5F3316B30

#### Trichotichnus (Trichotichnus) noctuabundus

Habu, 1954

A8FD68FC-9FF4-5FC3-90D2-7E4D97CC57F0

#### Trichotichnus (Trichotichnus) vespertinus

Habu, 1954

94502673-DA70-5F27-A717-DC88EBAE463A

#### Trichotichnus (Trichotichnus) vicinus

(Tschitschérine, 1897)

39D32AAA-D6FF-5E77-9091-F23A796C86E9

## Identification Keys

### Key to the Korean species of the genus *Trichotichnus*

**Table:** 

1	Frontal impression deep throughout, clearly reaching margin of eye; ligula narrow, not widened at apex; paraglossa separated from ligula by narrow notch (subgenusBottchrus Jedlička); median lobe of aedeagus with apical capitulum, internal sac in right lateral view (Fig. 2F) large hook-shaped	T. (B.) nanus Habu
–	Frontal impression becoming shallow near eye; ligula narrow or widened at apex; paraglossa separated from ligula by narrow or wide notch	2
2	Paraglossa wider, notch between ligula narrow; ligula not widened apically, parallel-sided or slightly narrowed before apex; elytral marginal umbilicate series not continuous; abdominal sternite VII of male with two pairs of setae (subgenusIridessus Bates); head and pronotum reddish brown or orange; basal angle of pronotum gently rounded	T. (I.) lucidus (Morawitz)
–	Paraglossa narrower, notch between ligula wide; ligula widened at apex or almost parallel-sided; abdominal sternite VII of male with one or two pairs of setae (subgenusTrichotichnus Morawitz)	3
3	Head and pronotum reddish brown or orange; basal angle of pronotum angulate	T. (T.) longitarsis Morawitz
–	Head and pronotum black or reddish black; basal angle of pronotum angulate or narrowly rounded	4
4	Metepisternum a little longer than wide, ML/MW = less than 1.15; pronotum cordate, surface clearly punctate, somewhat sparsely punctate in disc	T. (T.) coruscus (Tschitschérine)
–	Metepisternum distinctly longer than wide, ML/MW = more than 1.15; pronotum more or less punctate	5
5	Apical margin of abdominal sternite Ⅶ with a pair of setae in male and two pairs of setae in female; protibia longitudinally sulcate on dorsal side	6
–	Apical margin of abdominal sternite Ⅶ with two pairs of setae in male and female; protibia not longitudinally sulcate on dorsal side	7
6	Basal angle of pronotum narrowly rounded	T. (T.) noctuabundus Habu
–	Basal angle of pronotum angulate, protruding laterally	T. (T.) vespertinus Habu
7	Basal angle of pronotum narrowly rounded	T. (T.) miser (Tschitschérine)
–	Basal angle of pronotum angulate, more or less protruding laterally	8
8	Head wider, PW/HW = less than 1.41; proepisternum with faint punctures; length of metatarsus distinctly shorter than head, metatarsus/HW = less than 0.80; body length = 6.2–9.0 mm	T. (T.) congruus Motschulsky
–	Head narrower, PW/HW = more than 1.41; proepisternum with faint punctures anteriorly or nearly smooth; length of metatarsus slightly shorter than head, metatarsus/HW = more than 0.80	9
9	locProepisternum with faint punctures anteriorly; elytra narrower, EW/PW = 1.14–1.24; internal sac of aedeagus with long, peg-shaped sclerotized part; larger species, body length = 8.6–10.0 mm	T. (T.) nishioi Habu
–	Proepisternum nearly smooth; elytra wider, EW/PW = 1.25–1.33; internal sac of aedeagus without sclerotized part; smaller species, body length = 7.5–8.5 mm	T. (T.) vicinus (Tschitschérine)

The presented key is mainly based on Habu (1973) and Kataev (2020). The character of T. (T.) coruscus is based on the description of its junior synonym, *T.sachalinensis* by Habu (1954b), and T. (T.) vicinus is based on the original description and the description of its junior synonym, *T.pseudocongruus* and *T.iridulus* by Ito (2001) and Ito (2014), respectively.

## Supplementary Material

XML Treatment for Trichotichnus

XML Treatment for Trichotichnus (Bottchrus) nanus

XML Treatment for Trichotichnus (Trichotichnus) miser

XML Treatment for Trichotichnus (Trichotichnus) vespertinus

XML Treatment for Trichotichnus (Iridessus) lucidus

XML Treatment for Trichotichnus (Trichotichnus) longitarsis

XML Treatment for Trichotichnus (Trichotichnus) nishioi

XML Treatment for Trichotichnus (Trichotichnus) noctuabundus

XML Treatment for Trichotichnus (Bottchrus) nanus spinulosus

XML Treatment for Trichotichnus (Iridessus) lucidus

XML Treatment for Trichotichnus (Trichotichnus) congruus

XML Treatment for Trichotichnus (Trichotichnus) coruscus coruscus

XML Treatment for Trichotichnus (Trichotichnus) longitarsis

XML Treatment for Trichotichnus (Trichotichnus) miser

XML Treatment for Trichotichnus (Trichotichnus) nishioi

XML Treatment for Trichotichnus (Trichotichnus) noctuabundus

XML Treatment for Trichotichnus (Trichotichnus) vespertinus

XML Treatment for Trichotichnus (Trichotichnus) vicinus
